# Penetratin an Old Player in the Field of Cell-Penetrating Peptides Is in New Custom—Effect of Aromatic Non-Natural Amino Acid Substitutions

**DOI:** 10.3390/pharmaceutics18050555

**Published:** 2026-04-30

**Authors:** Dóra Soltész, Ildikó Szabó, Viktor Farkas, Nikolett Borók, Tamás Visnovitz, Dorina Lenzinger, Fülöp Károly Grébecz, Szilvia Bősze, Zoltán Bánóczi

**Affiliations:** 1 Department of Organic Chemistry, Institute of Chemistry, Faculty of Science, ELTE Eötvös Loránd University, Pázmány Péter Sétány 1/A, 1117 Budapest, Hungary; 2 Hevesy György PhD School of Chemistry, Institute of Chemistry, ELTE Eötvös Loránd University, Pázmány Péter Sétány 1/A, 1117 Budapest, Hungary; 3 HUN-REN-ELTE Research Group of Peptide Chemistry, 1117 Budapest, Hungary; 4 HUN-REN-ELTE Protein Modeling Research Group, 1117 Budapest, Hungary; 5 Department of Genetics, Cell- and Immunobiology, Faculty of Medicine, Semmelweis University, Nagyvárad tér 4, 1089 Budapest, Hungary; 6 Department of Plant Physiology and Molecular Plant Biology, ELTE Eötvös Loránd University, Pázmány Péter Sétány 1/c, 1117 Budapest, Hungary

**Keywords:** penetratin, cell-penetrating peptide, CPP, cellular uptake

## Abstract

**Background/Objectives**: Investigating the modified derivatives of known cell-penetrating peptides can highlight the important residues in the peptide sequence and help understand the cellular uptake mechanism better. Moreover, comparing peptides with different fluorescent-dye positions can highlight the importance of the conjugation site. Earlier, it was demonstrated that the fluorescence quencher 4-((4-(dimethylamino)phenyl)azo)benzoyl (Dabcyl) group can enhance the internalization efficiency of highly cationic oligoarginine peptides. However, its effect in the case of arginine-rich penetratin, a secondary amphipathic cationic CPP, remains undiscovered. **Methods**: Here, several penetratin derivatives were studied in which the aromatic residues were substituted and the effect of Dabcyl modification was also studied on the cellular uptake of peptides by flow cytometry. **Results**: The triple Nal-substituted penetratin and dodeca-penetratin with N-terminally positioned carboxyfluoresein (Cf) dye demonstrated remarkable internalization efficiency compared to penetratin. Moreover, almost all the Dabcyl-modified peptides were superior to penetratin except two peptides with C-terminal Cf-labelling. This result highlights the importance of the structure of the conjugate. The position of the cargo molecule may have a high impact on internalization ability. The relatively low cellular uptake of the Trp48 residue-substituted Dabcyl-Pen12 points to the importance of this residue in the cellular uptake of dodeca-penetratin. The confocal microscopic studies revealed that, besides the greater penetration efficiency of Dabcyl penetratin derivatives, these peptides enter the cytoplasm of cells in an increased manner. **Conclusions**: We identified several intriguing derivatives and expanded the applicability of Dabcyl, while also highlighting its limitations. Additionally, the critical role of Trp48 in the penetratin sequence was reaffirmed, along with the importance of the fluorescent molecule’s position.

## 1. Introduction

The field of cell-penetrating peptides (CPPs) is a rapidly growing research area with many potential therapeutic and diagnostic applications attributable to the fact that these small peptides can be used as delivery vehicles to transport diverse cargos (e.g., small molecules, peptides, proteins, oligonucleotides) into cells [1,2,3]. The major complications concerning the use of CPPs are their insufficient efficiency, lack of selectivity (unless there is a targeting part in the construct), poor stability and endosomal entrapment [4]. This last weakness of peptides can be avoided, e.g., by endosomal escape enhancing domains [5,6] or prompting direct translocation through the cell membrane, e.g., using 4-((4-(dimethylamino)phenyl)azo)benzoyl (Dabcyl) group [7] as internalization enhancer, whereas the stability of peptides can be improved, e.g., with the use of non-natural amino acids [8].

The Dabcyl group is routinely used as a fluorescence quencher chromophore in Förster-type resonance energy transfer (FRET)-based assays [9]. However, it was demonstrated that its coupling to linear or cyclic oligoarginines can enhance their internalization [7,10,11,12,13]; moreover, the surface modification of nanoparticles with numerous Dabcyl molecules improves their cellular uptake [10].

Penetratin (R^43^QIKIWFQNRRMKWKK^58^) is a widely known cell-penetrating peptide; its structure, mechanism of internalization, and related derivatives have been thoroughly investigated. The 16-mer peptide originates from the third helix of the Antennapedia homeodomain of *Drosophila melanogaster*, and it was found to effectively translocate into rat striatal nerve cells like the parent 60-mer peptide, although this latter showed enhanced translocation possibly due to the supporting two other helices [11]. Penetratin is a member of the secondary amphipathic cationic CPPs, and it adopts a partial alpha-helical structure near membrane lipids at low peptide/lipid ratios and low surface charge density [11,12,13]. Interestingly, the observed helical secondary structure of penetratin is not mandatory for its cellular uptake based on the results obtained with Pro substituted non-helical derivatives [14].

There have been several studies with different sequence variants of penetratin to clarify the amino acid residues which are the major contributors to its efficient cellular uptake. It was demonstrated that changing the two tryptophans (Trp) to phenylalanine (Phe) reduces the cellular uptake, although this derivative has a more pronounced, elongated alpha-helical structure than the penetratin [11,15,16,17]. In contrast, Thoren et al. found no significant difference in the internalization of the two peptides during confocal laser scanning microscopy [18]. Moreover, it was suggested that the phenylalanine residue is not essential for internalization, based on alanine-scanning studies [19]. This thorough alanine scanning also revealed that the tryptophan near the C-terminal of the peptide (Trp56) might be more indispensable than the Trp residue near the N-terminal (Trp48) which can be changed to alanine with a less decrease in cellular uptake. Opposed to this, Drin et al. found that the derivative containing alanine as a substitute of Trp56 was slightly better in internalization than the derivative where the Trp48 was changed to Ala (although both peptides were inferior to the parent peptide) [20]. This contradiction may arise from differences in cell lines and internalization assays used by the research groups. Recently, we gave evidence that replacing Trp56 with aza-glycine did not reduce, but rather increased the cellular entry of the 15-mer (methionine-lacking) penetratin, while changing Trp48 to aza-glycine or glycine severely hindered uptake into A-431 cells [21]. Besides this, it was revealed that the N-terminally located Trp has a significant role in the endocytosis-independent internalization of the peptide [22,23]; moreover, this Trp residue is more conserved among homeodomains than the C-terminal Trp [24]. Additionally, it was also demonstrated that amino acid deletion from the C-terminal is less tolerated than deletion from the N-terminal, and all basic residues except the arginine at the N-terminal play an important role in the cell penetration [19]. Furthermore, cyclization strongly impedes the cellular uptake since a cyclic penetratin derivative showed very poor internalization [19].

Due to the possible problems arising from incorporating methionine in the sequence, omitting this amino acid is quite preferable, and according to Szabó et al. the deletion of this residue does not influence the translocation ability of penetratin [1]. Although the shortest, minimal sequence originating from penetratin that can effectively penetrate HeLa cells in its biotinylated form consist of the RRMKWKK residues (named P7), this peptide was unable to carry its cargo peptide into the cytosol [25]. The authors supposed that the P7 peptide is trapped in endocytic vesicles, because it is unable to effectively interact with the lipid membrane and disrupt its structure in order to escape. Czajlik et al. designed and investigated a shortened penetratin peptide, named dodeca-penetratin, in which the Gln50-Asn51-Arg52-Met53 residues are deleted. This derivative showed similar cellular uptake as penetratin on several cell lines [15,16,17]; thus, it seems reasonable to study the derivatives of this shortened analogue also.

3-(2-naphthyl)-L-alanine (2-Nal, Nal) is a hydrophobic non-natural amino acid which was studied as a non-amphipathic Trp or Phe alternative in peptides [26,27,28]. Although the size of the aromatic side chain in case of Nal and of Trp is similar, the former is unable to form hydrogen bonds and possibly inserts deeper into the membrane. Nonetheless, due to its non-proteinogenic nature, it might have a stability increasing effect in peptides. In the literature, there are noteworthy examples of the utilization of Nal. Indolicin is a 13-mer hydrophobic antimicrobial peptide isolated from bovine neutrophils, which has five Trp residues [29]. It was demonstrated that replacing all Trp residues with Nal enhances the antibacterial and hemolytic activities of the parent peptide [26]. Similarly, in the case of another antibacterial peptide, the 15-mer bovine lactoferrin peptide, which contains two Trp amino acids, the replacement of these residues with Nal leads to more potent antibacterial activity [27]. In a cyclic arginine-rich peptide (cyclo(FFRRRRQ)), changing the second Phe residue to 2-Nal increased the cellular uptake by six times on MCF-7 cells [28].

L-1,2,3,4-tetrahydroisoquinoline-3-carboxylic acid (TIC) is another unnatural α-amino acid with a highly rigid, aromatic structure, which can be used as a constrained Phe analogue or a hydrophobic Pro substitute in peptide-based drugs and small molecules [30,31,32,33].

In this study, we investigated the different derivatives of penetratin and dodeca-penetratin where all the Trp and Phe residues or just one or two Trp residues were modified; in addition, we examined the effect of the Dabcyl molecule. We investigated the influence of the fluorescent molecule’s position and the peptide’s charge on the translocational activity of the 15-mer penetratin and its derivatives. We revealed several interesting derivatives among the 18 synthesized peptides, extended the applicability of Dabcyl while demonstrating its limitations; moreover, the importance of Trp48 in the sequence of penetratin was highlighted again, similarly to the significance of the position of the fluorescent molecule.

## 2. Materials and Methods

### 2.1. Synthesis of Peptides

The peptides were synthesized manually by solid-phase peptide synthesis (SPPS) on Rink amide MBHA resin, using the Fmoc/^t^Bu strategy. The side chain of arginine was protected with 2,2,4,6,7-pentamethyldihydrobenzofuran-5-sulfonyl (Pbf), while in case of lysine, the tert-butyloxycarbonyl (Boc) group was used. Triphenylmethyl (Trt) group was applied to protect the side chains of asparagine and glutamine. The α-amino group of amino acids were protected with Fmoc group, which was removed by the solution of 2% 1,8-diazabicycloundec-7-ene (DBU, Fluka (Buchs, Switzerland)) and 2% piperidine in dimethyl formamide (DMF, Molar Chemicals LTD (Budapest, Hungary)). The cleavage lasted 2 + 2 + 5 + 10 min according to the standard protocol. Extensive washing (8 × 1 min) was performed after the Fmoc-removal step. The coupling of the amino acid derivatives was carried out with N,N′-diisopropylcarbodiimide (DIC, IRIS Biotech GmbH (Marktredwitz, Germany)) and ethyl (hydroxyimino)cyanoacetate (OxymaPure, Sigma-Aldrich (Budapest, Hungary)) coupling reagents (three equimolar excess of each and the amino acid also) in DMF at room temperature (RT) for 60 min. Following the coupling, the resin was washed with DMF (2 × 1 min) and dichloromethane (DCM, Molar Chemicals LTD (Budapest, Hungary)) (3 × 1 min). To assess the success of the coupling, the Kaiser test was performed; if negative (successful coupling), the procedure was repeated until the given peptide sequence was complete; if positive, the procedure was repeated with the given amino acid. The removal of the last Fmoc group was followed by the attachment of Dabcyl (AAT BioQuest (Pleasanton, CA, USA)) or 5(6)-carboxyfluorescein (Cf, Sigma-Aldrich (Budapest, Hungary)) to the N-terminal amino acid on the resin using DIC/OxymaPure coupling reagents, whereas in case of the N-terminally acetylated peptides, DMF solution containing acetic acid anhydride and DIPEA (Fluka (Buchs, Switzerland)) (three times excess) was used. In case of peptides where the fluorescent molecule was coupled to a C-terminal Lys side chain, this residue was protected with the 1-(4,4-dimethyl-2,6-dioxocyclohex-1-ylidene)ethyl (Dde) group to permit its selective removal at the end of the solid-phase synthesis (following N-terminal modification). After using a solution of 2% hydrazine in DMF (2 + 2 + 5 + 10 min) for Dde removal, the 5(6)-carboxyfluorescein (Cf) was coupled to the liberated ε-amino group of the C-terminal Lys. The peptides were cleaved from the resin with 5 mL TFA (Sigma-Aldrich (Budapest, Hungary)) containing 0.365 g phenol (Sigma-Aldrich (Budapest, Hungary)), 0.25 mL distilled water, 0.25 mL thioanisole (Fluka (Buchs, Switzerland) and 0.125 mL 1,2-ethanedithiol (Sigma-Aldrich (Budapest, Hungary)) as scavengers. The obtained crude products were precipitated by dry diethyl-ether (Molar Chemicals LTD (Budapest, Hungary)), dissolved in 10% acetic acid (Molar Chemicals LTD (Budapest, Hungary)), lyophilized and subsequently purified by semi-preparative RP-HPLC. The purified compounds were characterized by analytical RP-HPLC and ESI-MS.

All amino acids and Rink amide MBHA resin were ordered form IRIS Biotech GmbH (Marktredwitz, Germany).

### 2.2. Determination of the Cellular Uptake by Flow Cytometry

For the examination of the internalization of the peptides, 10^5^ cultured EBC-1 or A-431 cells per well were plated on 24-well plates. The cells were incubated for 24 h at 37 °C then they were treated with peptides at different concentrations for 90 min in a serum-free medium. In the negative control, the cells were treated with a serum-free medium. After the incubation, the peptide solutions were removed and 100 µL trypsin (0.25%) was added for 5–9 min to remove membrane-bound peptides and detach the adherent cells from the plates. The activity of trypsin was terminated by the addition of 800 µL HPMI buffer (glucose, NaHCO_3_, NaCl, HEPES, KCl, MgCl_2_, CaCl_2_, Na_2_HPO_4_∙2H_2_O) containing 10% fetal bovine serum, and the cells were transferred from the plates to FACS-tubes for measurement. The cells were centrifuged at 216× *g* at 4 °C for 5 min and the supernatant was removed. The cells were resuspended in 250 µL HPMI, and the fluorescence intensity of the cells was quantified by flow cytometry (BD LSR II, BD Bioscience, San Jose, CA, USA). The data were analyzed with FACSDiva 5.0 software (BD Bioscience, San Jose, CA, USA). All reagents were ordered from Sigma-Aldrich (Budapest, Hungary).

The effect of the inhibitors was studied following the above-mentioned protocol, but cells were pretreated with an inhibitor for 30 min, following treatment with a peptide at 5 µM. The cells were then incubated for 90 min at 37 °C. Macropinocytosis was inhibited using 5-(N-ethyl-N-isopropyl)amiloride (EIPA) [34], the clathrin-mediated endocytosis was prevented with chlorpromazine (CPZ, TCI Chemicals, Tokyo, Japan) [35], methyl-cyclodextrin (CyD, TCI Chemicals, Tokyo, Japan) was applied for the inhibition of caveolae/lipid raft-mediated endocytosis [36], colchicine (COL, Sigma-Aldrich (Budapest, Hungary) was used to ascertain the role of microtubules and, thus, the importance of pinocytosis [37] and the combination of sodium azide and 2-deoxy-glucose (NaN_3_/DOG, Sigma-Aldrich (Budapest, Hungary) prevented ATP production in cells, thereby revealing the role of energy-independent internalization pathways [38].

Gating strategy: Flow cytometric analysis of cellular uptake and viability. Human A431 and EBC-1 cells were treated with the indicated Cf-labelled compounds under the conditions described above and subsequently harvested for flow cytometric analysis. After exclusion of debris and selection of the main cell population based on forward and side scatter characteristics, propidium iodide (PI) was used for viability discrimination. PI was added to cell samples immediately before acquisition at a final concentration of 10 µg/mL, and the PI-negative population was defined as the viable cell population, whereas PI-positive events were considered non-viable and excluded from further analysis. Medium-treated cells served as negative controls for setting the Cf-negative gate. Cellular uptake was quantified within the viable cell population, and the percentage of Cf-positive cells was determined from the corresponding histograms.

### 2.3. In Vitro Intracellular Localization Using Confocal Laser Scanning Microscopy (CLSM)

A-431 cells were seeded in complete medium in 24-well cell culture plates (Greiner Bio-One, Kremsmünster, Austria) with cover glasses (Assistant) for 24 h before the experiment. The cells were seeded at a density of 10^5^ cells per 1 mL per well onto cover glasses and incubated for 24 h. Cells were then treated with penetratin derivatives at concentrations of 12.5 for 90 min in ICM. According to the manufacturer’s instructions, lysosomes were labelled with LysoTracker Deep Red (ThermoFisher, Waltham, MA, USA), and Hoechst 33342 (Sigma-Aldrich, Budapest, Hungary) was used to stain the nuclei. Between staining steps, cells were washed three times with ICM. After two washes with PBS, the cells were fixed with 4% PFA for 20 min at 37 °C. They were then washed three times with PBS and twice with distilled water. Cover glasses were mounted onto microscope slides (VWR, Debrecen, Hungary) using Mowiol 4–88 mounting medium (Sigma-Aldrich (Budapest, Hungary)). Confocal microscopy was performed with a Leica TCS SP8 Lightning Confocal Laser Scanning Microscope (Leica, Wetzlar, Germany) equipped with a 63× oil objective as described in Bato et al. Image processing was performed using ImageJ software (https://imagej.net/ij/download.html, accessed on 12 December 2024).

### 2.4. Statistical Analysis

The results of the uptake studies are indicated as the mean value and standard deviation. Student’s *t* test was used for the statistical analysis. Results with *p* values < 0.05 were regarded as statistically significant.

## 3. Results

### 3.1. Synthesis of Peptides

According to previous results, the methionine residue of penetratin is dispensable and omitting it makes the synthesis more simple and convenient [1]; thus, we prepared the 15-mer penetratin (RQIKIWFQNRRKWKK) without the Met residue refer to it as penetratin (Pen) in the following sections.

**Figure 1:**
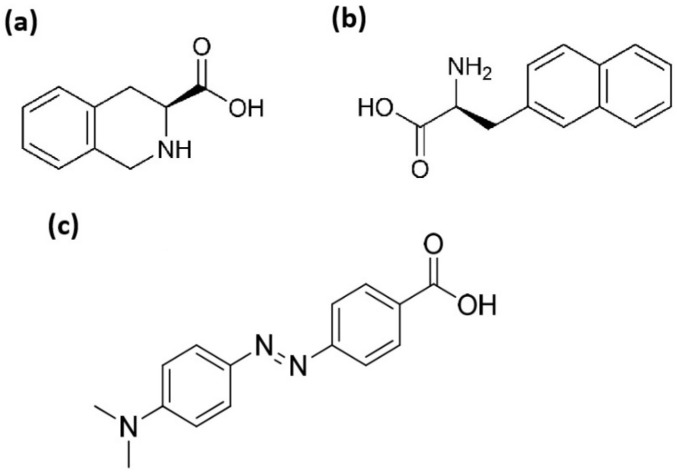
Structures of (**a**) L-1,2,3,4-tetrahydroisoquinoline-3-carboxylic acid (TIC), (**b**) 3-(2-naphthyl)-L-alanine (Nal) and (**c**) 4-((4-(dimethylamino)phenyl)azo)benzoic acid (Dabcyl).

In this study, we synthesized and examined several 15 and 12 amino acids long penetratin derivatives where the two tryptophan (Trp48 and Trp56) and one phenylalanine (Phe) residues were substituted by non-natural aromatic amino acids Nal and TIC (Figure 1). In addition, based on the advantageous effect of Dabcyl on the cellular uptake of oligoarginines [7,39,40,41,42], we were interested in the outcome of coupling Dabcyl to penetratin as an arginine-rich CPP and to some of its derivatives. The effect of the position of the fluorescent dye was also examined as a consequence of our earlier findings where the Dabcyl-R6K(Cf) peptide showed considerably higher cellular uptake than Cf-R6K(Dabcyl) on EBC-1 cells [41]. The expendability of the last C-terminal Lys side chain (Lys58) of dodeca-penetratin and, thus, the significance of the charge of the peptide was also investigated. The difference in the importance of the two Trp residues as demonstrated earlier [21] were revealed by studying analogues which contained only one Trp to 2-Nal substitution. All the peptides were synthesized manually by solid-phase peptide synthesis on Rink amide MBHA resin using Fmoc/*t*Bu strategy. The coupling reagents were DIC and OxymaPure, and for the cellular uptake quantification the fluorescent molecule 5(6)-carboxyfluorescein (Cf) was coupled either to the ε-amino group of an inserted or sequential lysine (Lys) at the C-terminus in solution, or to the N-terminus on solid phase such as Dabcyl group.

The purified peptides were characterized by analytical RP-HPLC and ESI-MS (Table 1). The analytical RP-HPLC chromatograms and MS spectra can be found in the Supplementary Materials (Figures S1–S44).

### 3.2. Cellular Uptake

The cellular uptake of peptides was determined using flow cytometry on EBC-1 human lung squamous carcinoma cells and on A-431 human skin squamous cancer cells. The solution of peptides were added to the cells at 5 or 10 µM concentration and cells were treated for 90 min at 37 °C.

The fluorescence intensity of peptide treated cells was corrected with the autofluorescence of untreated cells. The toxicity of the peptides was assessed by comparing the ratio of live and dead cells during the flow cytometry analysis. Based on this, none of the peptides showed cytotoxicity at the highest concentration used (Supplementary Materials Figures S58 and S59).

**Figure 2:**
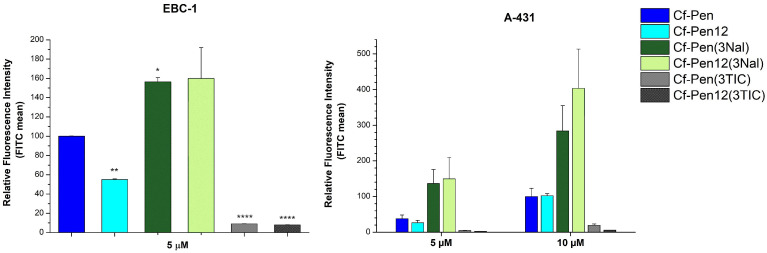
Comparing the cellular uptake efficiency of penetratin derivatives on EBC-1 and A-431 cells. The cells were treated with peptides at a concentration of 5 µM at 37 °C for 90 min. After trypsinization the fluorescence intensity of cells was studied using flow cytometry. The fluorescence intensities were normalized to the fluorescence intensity of cells that were treated with Cf-Pen(-Met) at 5 µM (EBC-1 cells) or at 10 µM (A-431 cells). Any significant difference from penetratin control of a given concentration was measured using Student’s *t* test. The asterisks show a significant difference between the control penetratin and the modified penetratin derivatives (* *p* < 0.05, ** *p* < 0.01, **** *p* < 0.0001).

At first we examined the influence of substitution of all three aromatic residues (Trp48, Trp56 ad Phe49) of penetratin derivatives by non-natural aromatic amino acids Nal and TIC. The internalization of prepared peptides (Table 1, row 1–6) was measured on both EBC-1 and A-431 cells at 5 µM concentration and on A-431 cells at 10 µM concentration (Figure 2). Interestingly, the dodeca-penetratin showed decreased cellular uptake compared to Cf-Pen at 5 µM on both cell lines (reduced efficiencies were 55.1% and 70.0% on EBC-1 and on A-431 cells, respectively), whereas the two peptides showed similar translocational activity on A-431 cells at 10 µM. Among the Cf-Pen derivatives, the naphthylalanine modified peptide Cf-Pen(3Nal) was better than the parent peptide on both cell lines at 5 µM concentration (1.6 and 3.6 times better internalization on EBC-1 and on A-431 cells, respectively) and on A-431 cells at 10 µM concentration (2.8 times greater uptake). Regarding the dodeca-penetratin derivatives, the Cf-Pen12(3Nal) peptide showed 2.9 and 5.6 times higher internalization efficiency than Cf-Pen12 at 5 µM on EBC-1 and A-431 cells, respectively. At 10 µM concentration on A-431 cells the Cf-Pen12(3Nal) peptide had ~4 times better cellular uptake compared to its counterpart. Remarkably, the Nal-modified peptides were even slightly (~1.2 times) better than octaarginine (Cf-R8) on EBC-1 cells (Figure 2). The tetrahydroisoquinoline derivatives (Cf-Pen(3TIC) and Cf-Pen12(3TIC)) had extremely low cellular uptake on both cell lines at 5 µM and on A-431 cells at 10 µM concentration.

**Figure 3:**
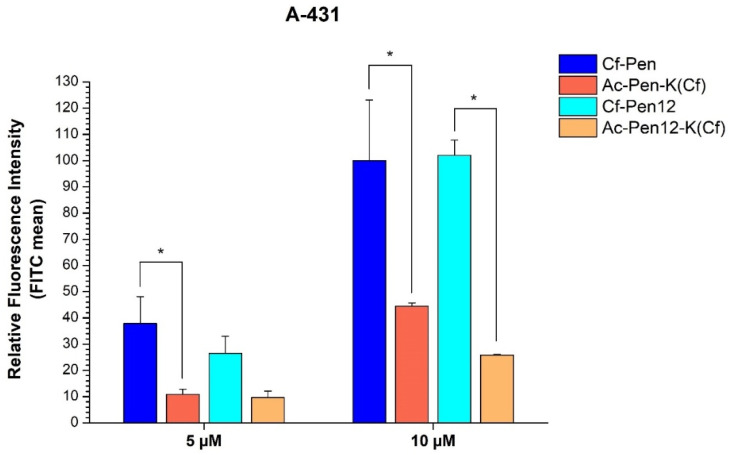
The effect of the position of Cf on the cellular uptake of penetratin(-Met) and dodeca-penetratin on A-431 cells at 5 µM and 10 µM concentrations. The fluorescence intensities were normalized to the fluorescence intensity of cells that were treated with Cf-Pen(-Met) at a concentration of 10 µM (100%). Any significant difference from the corresponding penetratin control (Cf-Pen or Cf-Pen12) of a given concentration was measured using Student’s *t* test (* *p* < 0.05).

In order to precisely examine the effect of Dabcyl on the cellular uptake of penetratin, N-terminally acetylated derivatives as controls were prepared where the Cf molecule was carried by a C-terminally inserted Lys residue (Ac-Pen-K(Cf) and Ac-Pen12-K(Cf)). Interestingly, when comparing these peptides with Cf-Pen and Cf-Pen12, it was observed that the position of the fluorescent molecule had a significant influence on the cellular uptake efficiency (Figure 3). Considering the 15-mer penetratin, the Ac-Pen-K(Cf) peptide showed decreased internalization on A-431 cells at both concentrations (reduction to 28.8% and to 44.5% of Cf-Pen at 5 µM and 10 µM, respectively). The same relation holds true for the dodeca-penetratin analogues Cf-Pen12 and Ac-Pen12-K(Cf) also (here the difference in efficiency declined to 36.3% and to 25.3% of Cf-Pen12 at 5 µM and 10 µM, respectively, and it only reaches statistical significance at 10 µM concentration).

**Figure 4:**
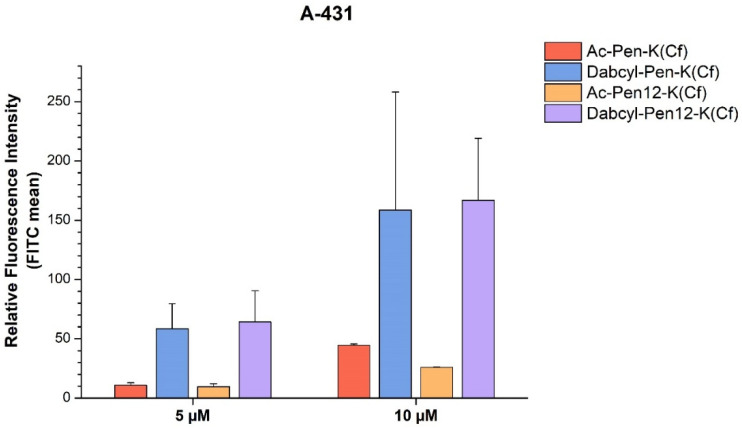
The effect of Dabcyl on the cellular uptake of penetratin(-Met) and dodeca-penetratin on A-431 cells at 5 and 10 µM concentration. The fluorescence intensities were normalized to the fluorescence intensity of cells that were treated with Cf-Pen(-Met) at a concentration of 10 µM (100%). Any significant difference from the corresponding penetratin control (Ac-Pen(-Met)-K(Cf) or Ac-Pen12-K(Cf)) of a given concentration was measured using Student’s *t* test. No significant difference was found.

With regard to the Dabcyl-modified penetratin and dodeca-penetratin derivatives, both had greatly enhanced internalization compared to the acetylated control peptides (Figure 4). The Dabcyl-Pen-K(Cf) peptide was 5.4 and 3.6 times more efficient as a CPP than Ac-Pen-K(Cf) on A-431 cells at 5 and 10 µM, respectively, whereas Dabcyl-Pen12-K(Cf) showed 6.7 and 6.5 times greater cellular uptake than Ac-Pen12-K(Cf) at 5 µM and 10 µM concentration. The Dabcyl-modified peptides were also ~1.5 times superior to Cf-Pen at both concentrations.

**Figure 5:**
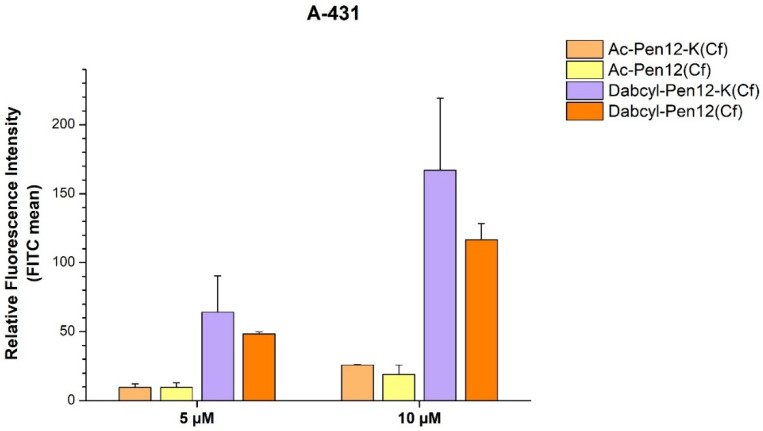
The effect of removed positive charge on the cellular uptake of acetylated and Dabcyl-modified dodeca-penetratin on A-431 cells at 5 and 10 µM concentration. The fluorescence intensities were normalized to the fluorescence intensity of cells that were treated with Cf-Pen(-Met) at a concentration of 10 µM (100%). Any significant difference from the corresponding penetratin control (Ac-Pen12-K(Cf) or Dabcyl-Pen12-K(Cf)) of a given concentration was measured using Student’s *t* test. No significant difference was found.

To determine the effect of the reduced charge of dodeca-penetratin by coupling the fluorescent molecule Cf to the side chain of the last C-terminal Lys (Lys58), we prepared the peptides presented in lines 11–12 of Table 1. In case of the acetylated peptide at 10 µM and the Dabcyl-modified peptides at both concentration the deletion of the amino group of Lys58 caused a reduction in the peptide translocation into A-431 cells (Figure 5). The difference between Ac-Pen12-K(Cf) and Ac-Pen12(Cf) seems negligible at 5 µM (0.38% decrease), but at 10 µM the internalization is reduced by 26.3% when the positively charged side chain of Lys58 is lacking. With regard to the Dabcyl-modified peptides, the decrease in efficiency was similar at both concentrations (24.7% and 30.2% reduction at 5 and 10 µM, respectively), although no statistical significance was found concerning both pair of peptides at either concentration. The Dabcyl-Pen12(Cf) peptide demonstrated a slightly increased cellular uptake compared to Cf-Pen(-Met).

**Figure 6:**
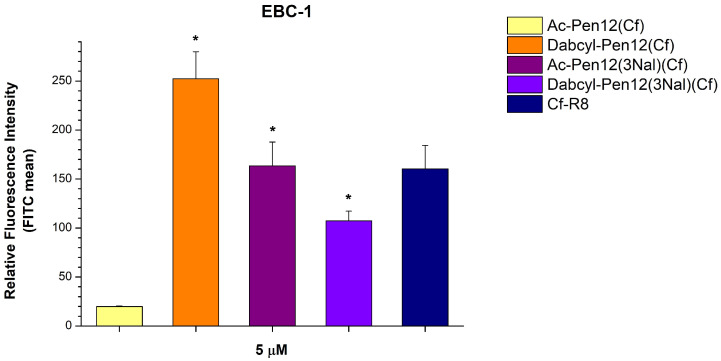
Cellular uptake efficiencies of several dodeca-penetratin derivatives on EBC-1 cells at 5 µM concentration. The fluorescence intensities were normalized to the fluorescence intensity of cells that were treated with Cf-Pen(-Met) at a concentration of 5 µM (100%). Any significant difference from Ac-Pen12(Cf) was measured using Student’s *t* test (* *p* < 0.05).

**Figure 7:**
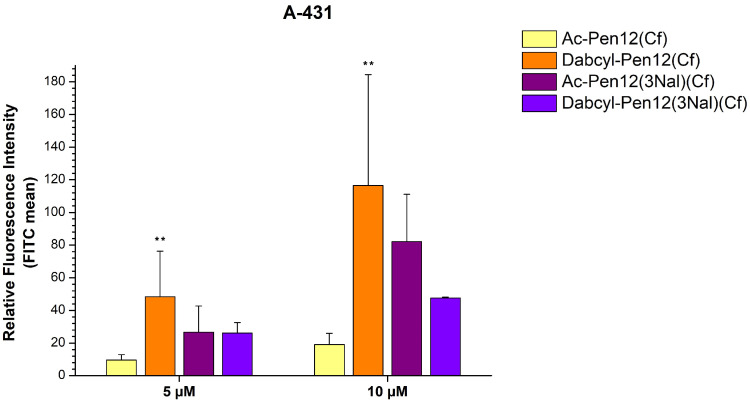
Cellular uptake efficiencies of several dodeca-penetratin derivatives on A-431 cells at 5 and 10 µM concentrations. The fluorescence intensities were normalized to the fluorescence intensity of cells that were treated with Cf-Pen(-Met) at a concentration of 10 µM (100%). Any significant difference from Ac-Pen12(Cf) was measured using Student’s *t* test (** *p* < 0.01).

Considering the efficient Nal containing peptides Cf-Pen(3Nal) and Cf-Pen12(3Nal) (Figure 2) and the fact that Dabcyl-Pen12(Cf) was much better at cellular entry than Ac-Pen12-K(Cf) (Figure 5), suggesting that the Dabcyl modification can overcome the reduction in cell penetration efficiency caused by the loss of a positive charge, we further examined some Pen12(Cf) derivatives with Dabcyl and/or Nal modifications (lines 13–14 in Table 1). Regarding the triple Nal-modified peptides, Dabcyl-Pen12(3Nal)(Cf) showed lower cellular uptake than Dabcyl-Pen12(Cf) and also than its acetylated version Ac-Pen12(3Nal)(Cf) on EBC-1 cells at 5 µM concentration (57.5% and 34.4% decrease in internalization, respectively), although it had 5.4 times greater uptake than Ac-Pen12(Cf) (Figure 6). The same relation of efficiencies was observed on A-431 cells at 10 µM concentration (Dabcyl-Pen12(3Nal)(Cf) demonstrated 59.2% and 42.1% reduction in cellular uptake compared to Dabcyl-Pen12(Cf)and Ac-Pen12(3Nal)(Cf), respectively, and 2.50 times higher internalization than Ac-Pen12(Cf)), whereas at 5 µM the Ac-Pen12(3Nal)-Cf peptide had similar translocational activity as its Dabcyl counterpart (Figure 7). Comparing all derivatives to Ac-Pen12(Cf), they were all significantly better on EBC-1 cells at 5 µM, but on A-431 cells, statistical significance was reached only in case of Dabcyl-Pen12(Cf). The discrepancy between Ac-Pen12(3Nal) and Dabcyl-Pen12(3Nal) was not statistically significant on either of the cell lines at the examined concentrations. It is also important to highlight that the cellular uptake of Dabcyl-Pen12(Cf) on EBC-1 cells was even greater than that of Cf-R8, and this difference was found to be statistically significant.

**Figure 8:**
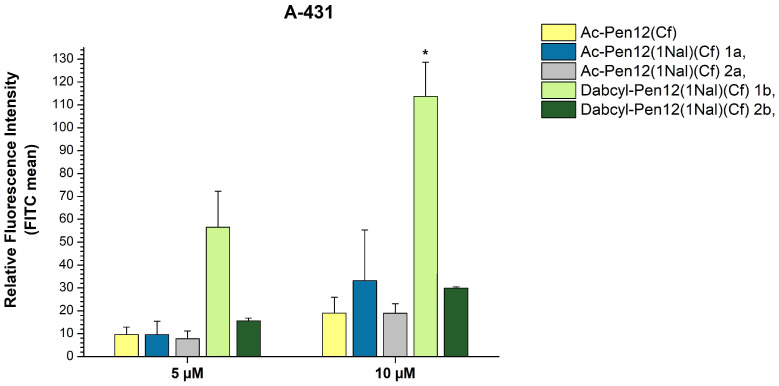
Cellular uptake efficiencies of several dodeca-penetratin derivatives on A-431 cells at 5 and 10 µM concentrations. The fluorescence intensities were normalized to the fluorescence intensity of cells that were treated with Cf-Pen at a concentration of 10 µM (100%). Any significant difference from Ac-Pen12(Cf) was measured using Student’s *t* test (* *p* < 0.05).

In order to ascertain the effects of changing only the N- or the C-terminal Trp to Nal, and to further investigate the influence of Dabcyl in case of these single Nal-modified penetratin derivatives, the peptides in line 15–18 of Table 1 were synthesized and their cellular uptake was measured on A-431 cells at 5 and 10 µM concentrations (Figure 8). The contrast revealed between the acetylated peptides with different substituted Trp residues (1a and 2a peptides) was slight at 5 µM (18.4% reduction in internalization when Trp48 was changed to Nal instead of Trp56), while at 10 µM it was noteworthy (42.9% decrease), but still statistically insignificant. On the other hand, between the Dabcyl-modified derivatives a truly remarkable disparity was observed (72.4% and 73.7% decline in internalization when Trp48 was changed to Nal instead of Trp56 at 5 and 10 µM concentration, respectively). Comparing all of the derivatives to their parent peptide Ac-Pen12(Cf), among the acetylated peptides no statistically significant difference in cellular uptake was found. Regarding the Dabcyl peptides, the Trp48 modified derivative (2b peptide) demonstrated a 1.6 times better uptake efficiency at both concentrations compared to the parent peptide, while the Trp56 modified analogue (1b peptide) was 5.9 and 6 times more effective than Ac-Pen12(Cf) at 5 and 10 µM, respectively. This derivative showed significantly greater cellular entry in comparison with all peptides mentioned at 10 µM concentration.

**Figure 9:**
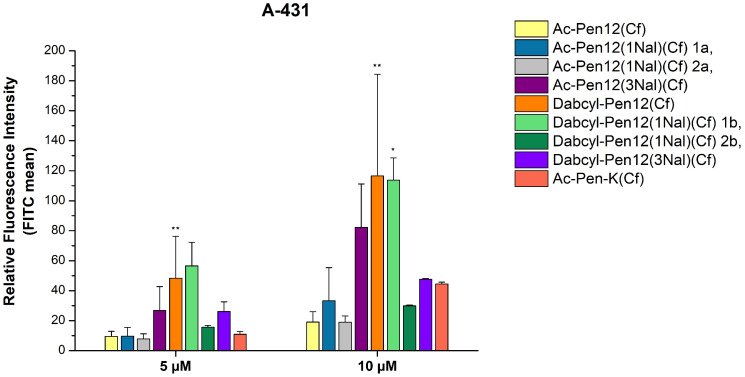
Cellular uptake efficiencies of all dodeca-penetratin derivatives on A-431 cells at 5 and 10 µM concentrations. The fluorescence intensities were normalized to the fluorescence intensity of cells that were treated with Cf-Pen(-Met) at a concentration of 10 µM (100%). Any significant difference from Ac-Pen12(Cf) was measured using Student’s *t* test (* *p* < 0.05, ** *p* < 0.01).

The comparison of all the Ac-Pen12(Cf) derivatives is visible in Figure 9. It is worth mentioning that in case of the acetylated peptides the triple Nal-modified analogue possesses the highest uptake efficiency at both concentrations, while in case of the Dabcyl peptides, Dabcyl-Pen12(3Nal) is only better than the single Nal-modified 2b derivative, since the 1b and the Trp-containing Dabcyl-Pen12(Cf) peptides showed greater internalization. Four peptides, namely Ac-Pen12(3Nal)(Cf), Dabcyl-Pen12(Cf), Dabcyl-Pen12(1Nal)(Cf) (1b) and Dabcyl-Pen12(3Nal)(Cf) had even greater translocational activity than Ac-Pen-K(Cf).

### 3.3. Examining the Mechanism of Cellular Entry

The mechanism of internalization of some selected penetratin derivatives were studied. A-431 cells were preincubated for 30 min with several endocytic inhibitors to reveal their effects on the cellular uptake of peptides. The inhibitors used were the following ones: 5-(N-ethyl-N-isopropyl)amiloride (EIPA) as an indirect macropinocytosis inhibitor [34]; chlorpromazine (CPZ) as the clathrin-mediated endocytosis inhibitor [35]; methyl-beta-cyclodextrin (mBCD) as the inhibitor of the lipid raft/caveolae-mediated endocytosis [36]; the pinocytosis inhibitor colchicine (COL) [37]; sodium azide (NaN_3_) and 2-deoxyglucose (DOG) to decrease cellular ATP generation by oxidative phosphorylation and by glycolysis, and thereby study the role of energy-independent internalization pathways [38].

**Figure 10:**
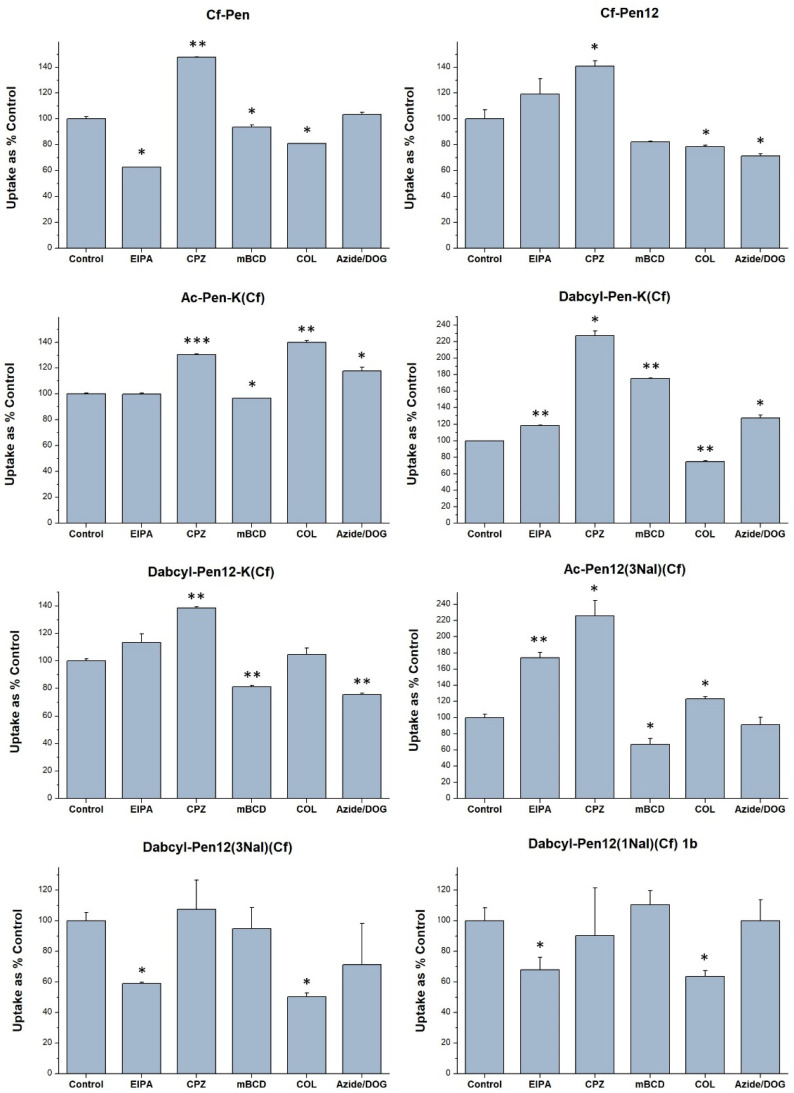
The effect of several endocytic inhibitors on the cellular internalization of chosen peptides. A-431 cells were pretreated with the inhibitors EIPA (50 µM), CPZ (30 µM), mBCD (2.5 mM), COL (5 mM), NaN_3_ (50 µM) and DOG (25 mM) for 30 min, then the cells were treated with the peptides (5 µM) for 90 min. Any significant difference from the control was measured using Student’s *t* test (* *p* < 0.05, ** *p* < 0.01, *** *p* < 0.001).

The results of the assay (Figure 10) indicated that EIPA decreased the cell penetration efficiency in case of Cf-Pen (to 62.8% of untreated control), Dabcyl-Pen12(3Nal)(Cf) (to 59.0%) and Dabcyl-Pen12(1Nal)(Cf) 1b (to 68.1%), and interestingly this inhibitor significantly increased the uptake of Dabcyl-Pen-K(Cf) and Ac-Pen12(3Nal)(Cf). Chlorpromazine (CPZ) markedly enhanced the cellular entry of all the derivatives examined except Dabcyl-Pen12(1Nal)(Cf) 1b, and the observed uptake improvement was noteworthy regarding Dabcyl-Pen-K(Cf) (to 227%). Methyl-beta-cyclodextrin slightly decreased the internalization of Cf-Pen12, Dabcyl-Pen12-K(Cf) and Ac-Pen12(3Nal)(Cf), whereas the uptake efficiency of Dabcyl-Pen-K(Cf) was even augmented when the cells were preincubated with mCBD (to 175.5%). Colchicine moderately impeded the translocational activity of Cf-Pen, Cf-Pen12 and Dabcyl-Pen-K(Cf), and this inhibitor had stronger effect in case of Dabcyl-Pen12(1Nal)(Cf) 1b and Dabcyl-Pen12(3Nal)(Cf) (reduction in fluorescence intensity to 63.6% and to 50.4% of controls, respectively). The internalization of Ac-Pen-K(Cf), Ac-Pen12(3Nal)(Cf) and Dabcyl-Pen12-K(Cf) was notably or slightly increased (to 139.8%, to 123.4% and to 104.7% of control, respectively) due to colchicine. The ATP-depleting reagents NaN_3_ and DOG decreased the internalization of all the dodeca-penetratin analogues except the Dabcyl-Pen12(1Nal)(Cf) 1b peptide.

### 3.4. Investigating the Intracellular Localization of Peptides

**Figure 11:**
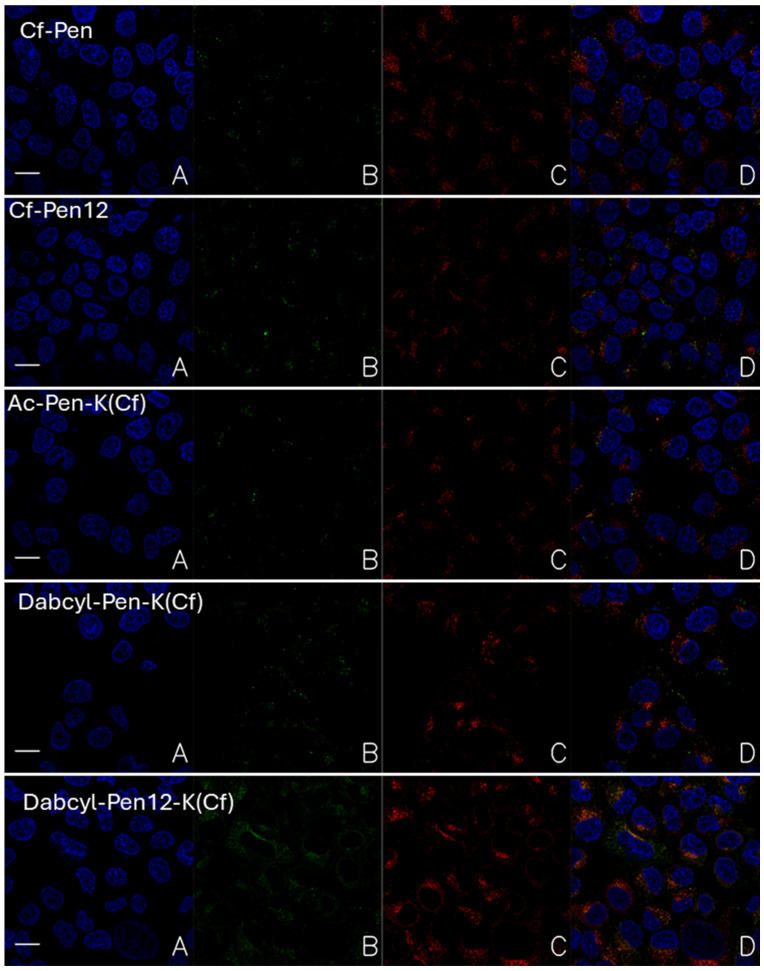
Internalization of peptides captured by confocal laser scanning microscopy using unprocessed images. Cells were incubated for 90 min with peptide (c = 12.5 µM). Nuclei were stained with Hoechst 33342, blue (**A**); Cf labelled peptide, green (**B**); lysosomes were labelled with LysoTracker Deep Red, red (**C**); merged channels (**D**). Cells were examined using a Leica SP8 microscope with an HC PL APO CS2 63×/1.40 OIL objective and the hybrid detector. The scale bar represents 20 µm.

To gain insight into the intracellular distribution of peptides Cf-Pen, Cf-Pen12, Ac-Pen-K(Cf), Dabcyl-Pen-K(Cf) and Dabcyl-Pen12-K(Cf), confocal laser scanning microscopy images were recorded on A-431 cells. Following peptide treatment (90 min incubation at c = 12.5 µM), the nuclei of cells and the lysosomes were stained with Hoechst 33342 and LysoTracker Deep Red, respectively, thereby the subcellular localization of peptides could be determined. The representative images are presented in Figure 11.

The intensity of internalized fluorescently labelled peptides showed the similar tendency which was observed by flow cytometry. The weakest fluorescent signal was detected when the cells were treated by Ac-Pen-K(Cf). The Cf labelled penetratin and its shorter derivative had higher fluorescent intensity (Cf-Pen and Cf-Pen12 respectively in Figure 11). The Dabcyl-modified derivatives (Dabcyl-Pen-K(Cf) and Dabcyl-Pen12-K(Cf) in Figure 11) showed enhanced fluorescence, and the Dabcyl-Pen12-K(Cf) was the best. Based on the colocalization of the nuclear staining and the fluorescent signal of peptides it was proved that they could not transport into the nucleus. All of the peptides were localized in the cytosol.

The examined peptides showed different degree of co-localization with lysosomes, which had correlation with the length of the peptide, the position of the Cf molecule and the presence of Dabcyl. The dodeca-penetratin derivatives Cf-Pen12 and Dabcyl-Pen12-K(Cf) showed higher co-localization in lysosomes than their 15-mer counterparts Cf-Pen and Dabcyl-Pen-K(Cf) (Figure 11). The position of the fluorescent molecule Cf also had an effect not only on the internalization efficiency of the peptide, but also the lysosomal co-localization. Penetratin with Cf on its N-terminal showed better cellular uptake and lower accumulation in lysosomes (Supplementary Materials Figures S45–S54 and Tables S1–S10, [43,44,45,46,47,48,49]).

Interestingly, Dabcyl-Pen-K(Cf) co-localized with lysosomes to a higher degree than its acetylated counterpart Ac-Pen-K(Cf), although the internalization efficiency of the Dabcyl peptide was superior.

In case of all peptides punctuate signal was detected which supposes the vesicular transport across the cell membrane. The Dabcyl-Pen12-K(Cf) peptides was the only exception because in its case intensive diffuse fluorescent signal was also recorded. Based on it we can say this is the only peptide which shows significant cytosolic distribution at 12.5 µM concentration. 

## 4. Discussion

It was demonstrated earlier that Dabcyl enhances the cellular entry of oligoarginines into cells [7,39,40,41,42]. Our results here extend the applicability of the Dabcyl group based on its internalization enhancer effect on the cationic amphipathic penetratin peptide. It is supposed that this group changes the interaction pattern of the peptides with the cell membrane and thus could increase the internalization. As penetratin has very a different interaction with the membrane, it is worth studying the extendibility of using Dabcyl as a cellular uptake enhancer. A dodeca-penetratin derivative has very similar properties as that of penetratin [15,16,17]; thus, we also used it in our structure–activity studies.

In these studies, we examined penetratin and dodeca-penetratin derivatives where one Trp or all of the aromatic residues were substituted with unnatural amino acids Nal and TIC. The combined effect of these modifications with the N-terminally conjugated Dabcyl was also investigated. As it is rarely studied, we examined the effect of the position of the Cf molecule.

The literature data suggest that the dodeca-penetratin, a shortened derivative of penetratin, enters cells with the same efficiency as the full-length peptide [15,16,17]. Contrasting these earlier results, our findings on EBC-1 and A-431 cells indicated the inferiority of the dodeca-penetratin at 5 µM concentration, but the peptides had similar internalization efficiency at 10 µM on A-431 cells (Figure 2). These findings may reveal that their internalization is dependent on the cell line. However, it should be mentioned that we used penetratin derivatives that do not contain methionine and we did not notice any difference in their internalization efficiency earlier [1]; it might be the reason of the observed dissimilarity. Additionally, the deletion of Arg52 in the sequence of Cf-Pen12 results in the absence of important cation-pi interactions, and this might impair the translocation of the peptide [50].

Among the triple modified peptides where the fluorescent molecule was attached to the N-terminal of the derivatives, the Nal-modified penetratin (Cf-Pen(3Nal)) and dodeca-penetratin (Cf-Pen12(3Nal)) proved to be remarkably good alternatives on both EBC-1 and A-431 cells, showing greater cellular uptake compared to the parent peptides (Figure 2). Regarding the TIC modification, it probably strongly disrupts the structure of the peptides by creating a loop-like section (a structural turn) in the middle of the sequences, thereby impairing the internalization. Additionally, the lower hydrophobicity of TIC compared to Trp and the non-planar structure might also play a role in the observed inefficiency of peptides. Interestingly, the electronic circular dichroism (ECD) studies performed with Pen12, Pen12(3Nal) and Pen12(3TIC) (Supplementary Materials, Table S11 and Figures S56–S58) revealed no striking structural difference between Pen12(3Nal) and Pen12(3TIC) in solution, except for the band at around 230 nm, which can be identified as an extensive aromatic signal contributed by the naphthyl group; both peptides had unordered/dynamic structure. Based on this, we can suggest that there is no strong solution phase structure–activity relationship in the case of the penetratin derivatives, as demonstrated earlier [14]. But the different amino acid composition may have strong influence on the structure of membrane bound peptide and on its possible interactions with the membrane.

As it was shown earlier in case of the Dabcyl-oligoarginine peptides, the Cf-R6K(Dabcyl) peptide was much less efficient than its counterpart Dabcyl-R6K(Cf), but in case of the tetra- and pentaarginine derivatives there were no significant differences between the internalization of the pairs with the same number of arginine residues [41]. Considering the 15-mer and 12-mer penetratin peptides, comparing Cf-Pen and Cf-Pen12 with Ac-Pen-K(Cf) and Ac-Pen12-K(Cf) on A-431 cells at 5 and 10 µM concentrations, it seems that the Cf molecule is highly preferred on the N-terminal of the peptide, and positioning it to the side chain of a C-terminally inserted Lys decreases the cellular uptake (Figure 3) along with the reduction of retention times, as the result of the lower hydrophobicity. This is consistent with earlier studies where the peptides having Cf at their N-terminus had a slightly more hydrophobic character in the case of oligoarginine derivatives with Dabcyl or 1-pyrene carboxylic acid (PCA) modification [41,51]. Here the increased hydrophobicity is accompanied by greater cell penetration efficiency just like in case of PCA-oligoarginines [51], while concerning the Dabcyl-R6(Cf) and Cf-R6(Dabcyl) peptides, the rise in hydrophobicity implied lower uptake [41]. To summarize, it seems like that the Cf group is preferred on the N-terminal of peptides unless Dabcyl is also present in the sequence. Investigating the effect of the position of the fluorescent molecule is usually not included in CPP studies, although it was shown that the fluorophore can have an impact on the peptide–membrane interactions, the cellular uptake and the conformation of the peptide [52,53]. Our examples serve to emphasize that the position of the fluorescent group can have a significant influence on the internalization efficiency of peptides. It also demonstrates that even the position of the cargo in a bioconjugate may have high influence on the cellular uptake and thus biological activity.

Related to the position of the Cf, the expendability of the last C-terminal Lys side chain was investigated by preparing acetylated and Dabcyl-modified dodeca-penetratin derivatives where Cf was attached to Lys58, thereby reducing the charge of the peptides by one (from +6 to +5). It is reasonable to assume that the shorter peptides with less charge would have slightly higher hydrophobic character and thereby greater retention times. The observed slight differences in cellular uptake between the acetylated derivatives at 5 and 10 µM on A-431 cells suggest that the side chain of Lys58 has a minor role in the uptake process of Ac-Pen12-K(Cf), and it is expendable to carry the fluorescent molecule (Figure 5). If we assume that the penetratin and dodeca-penetratin have similar behaviours, this somehow contradicts the findings of Drin et al. who pointed to the fact that the mutation of any charged residue in the sequence of the NBD-labelled 16-mer penetratin drastically diminishes the internalization of the peptide into K562 human leukemia cells [20]. In case of the Dabcyl peptides, the alteration of cellular uptake of Dabcyl-Pen12(Cf) and of Dabcyl-Pen12-K(Cf) is noteworthy but still statistically insignificant. The explanation of these result could be that the Dabcyl modification provides hydrophobicity to the peptides which is better balanced when the side chain of Lys58 is present.

The main outcome of this study is that it reinforces the unique cellular uptake enhancing ability of the Dabcyl group. Both Pen and Pen12 showed greater internalization with Dabcyl on their N-terminal (Figure 4); nevertheless, the effect of the combination of the Dabcyl and Nal modifications is less straightforward. Considering the Dabcyl-Pen12(Cf) and Dabcyl-Pen12(3Nal)(Cf) peptides, the latter highly hydrophobic derivative surprisingly exhibited markedly lower internalization efficiency than the former one, and also than Ac-Pen12(3Nal)(Cf). It was proved that penetratin is not hydrophobic enough to insert deeply into the membrane bilayer [12]. It can only perturb lipid acyl chains which are in close proximity and interact with it based on molecular dynamic simulation investigations [50]. Moreover, Balayssac et al. concluded that, in the case of penetratin and other homeoprotein-derived peptides, the depth of insertion into model micelles was not correlated with the efficiency of internalization into CHO cells [54]. With regard to all of these, it is reasonable to assume that the remarkably hydrophobic character of Dabcyl-Pen12(3Nal)(Cf) facilitates overly powerful and deep interactions with the cell membrane, leading to the peptide remaining binding to it. Additionally, the inability of the Nal side chain to form hydrogen bonds might contribute to the mentioned inferiority of this peptide to Dabcyl-Pen12(Cf).

Regarding the single Nal-modified dodeca-penetratin peptides, a common tendency was observed: the substitution of Trp56 was more favourable than changing Trp48. The difference in the analogues was more pronounced in the case of Dabcyl peptides. The more prominent role of Trp48 in cellular uptake was revealed earlier when changing Trp48 to azaGly or Gly in the sequence of the Met-lacking penetratin was quite disadvantageous, while changing Trp56 to azaGly even increased the internalization [21]. It was also demonstrated that Trp48 inserts deeper into the membrane than Trp56 and mediates bilayer perturbation, thereby playing a major role in the energy-independent uptake of the peptide [22,23,55].

The examination of the effect of several endocytosis inhibitors on the cellular uptake of selected penetratin derivatives on A-431 cells provided interesting results. The macropinocytosis inhibitor EIPA hampered the internalization of Cf-Pen, Dabcyl-Pen12(3Nal)(Cf) and Dabcyl-Pen12(1Nal)(Cf) 1b, while regarding the other peptides, it caused an increase (Cf-Pen12, Dabcyl-Pen-K(Cf), Dabcyl-Pen12-K(Cf) and Ac-Pen12(3Nal)(Cf)) or no alteration (Ac-Pen-K(Cf)) in cellular uptake. This is in harmony with some previous investigations which highlighted the role of macropinocytosis in the cellular uptake of penetratin [21,56,57], but in contrast with another study where penetratin was affected by EIPA only at 50 µM concentration [58]. The lack of decreased internalization when applying chlorpromazine point to the fact that clathrin-mediated endocytosis is not a significant cellular entry mode regarding the studied penetratin derivatives, and this is consistent with previous investigations by our research group concerning penetratin and its derivatives [21]. According to the literature, penetratin enter cells mainly through lipid raft/caveolae-mediated endocytosis, although it can also utilize the macropinocytic and the energy-independent routes [17,21,56]. Concerning our examined peptides, mBCD caused a moderate reduction in the internalization efficiency of the dodeca-penetratin derivatives Cf-Pen12, Dabcyl-Pen12-K(Cf) and Ac-Pen12(3Nal)(Cf) (to 82.2%, to 81.2% and to 66.8% of controls, respectively), while Cf-Pen, Ac-Pen-K(Cf) and Dabcyl-Pen12(3Nal)(Cf) were affected by this inhibitor only to a very small degree (few percent loss in uptake efficiency), and the uptake of Dabcyl-Pen-K(Cf) and Dabcyl-Pen12(1Nal)(Cf) 1b was even enhanced. These results are quite remarkable and are somewhat in contrast with earlier findings obtained by the utilization of mBCD to study its effects on the internalization of fluorescein labelled penetratin and dodeca-penetratin on HeLa, L929 and RAW cells [17]. In that study the inhibition caused by mBCD extended to the 15-mer penetratin too, unlike in our case, and the strength of inhibition was considerably greater. Colchicine negatively affected the Cf-Pen, Cf-Pen12, Dabcyl-Pen-K(Cf), Dabcyl-Pen12(3Nal)(Cf) and Dabcyl-Pen12(1Nal)(Cf) 1b peptides; consequently, pinocytosis is an endocytic pathway through which these peptides can enter A-431 cells. The role of the energy-independent cellular uptake was investigated by applying the energy metabolism inhibitors NaN_3_ and DOG. The results indicated that the internalization of almost all of the dodeca-penetratin derivatives is partly energy-dependent, while the 15-mer penetratin peptides and the Dabcyl-Pen12(1Nal)(Cf) 1b peptide can enter cells during energy-depleted conditions just as efficiently as without inhibition. This is another clear contradiction to earlier results where energy-depletion led to a marked reduction in cellular entry of the 16-mer penetratin and also dodeca-penetratin [17]. The above-mentioned disparities can derive from the different modes of endocytosis inhibition: in the studies of Letoha et al., the endocytotic cellular uptake was inhibited by incubating the cells with the peptides at 4 °C, which affects the fluidity of the membrane and can thereby have a negative influence on the degree of passive penetration [59].

Microscopic experiments have shown that penetratin and its shorter derivative mainly enter cells via some form of endocytosis, as we mainly detected a punctate distribution. The fluorescent signal of all peptides was localized in the cytosole, and no nuclear staining was observed conflicting the results of earlier studies, albeit this discrepancy can be attributed to the difference in cell types [11,16,17]. Increasing the concentration of peptides caused the appearance of a small extent of diffuse distribution at least in case of dodeca-penetratin. The presence of the Dabcyl group resulted in diffuse distribution in case of only the Dabcyl-Pen12-K(Cf) at 5 µM concentration. The increased concentration also resulted in similar distribution when Dabcyl-Pen(Cf) was used. Based on these findings we can say that the Dabcyl group not only enhances the internalization of penetratin, but it also increases the direct translocation or the release from the endosomes.

Concluding all these, it seems that the position of the Cf molecule, the length of the penetratin peptide chain (15-mer or 12-mer penetratin derivatives), the presence of the Dabcyl group and the cell line on which the uptake of peptides was studied all exert influence on the cellular uptake of the examined penetratin peptides.

## 5. Conclusions

This study investigated the effect of substituting the aromatic moieties in the sequence of the penetratin peptide and of its shortened derivatives; furthermore, the applicability of the penetration enhancer Dabcyl was expanded. The importance of the position of the fluorescent molecule and the relative significance of the two Trp residues in penetratin were also revealed.

Among the peptides with an N-terminally positioned Cf label, the triple Nal-modified penetratin and dodeca-penetratin demonstrated remarkable internalization efficiency compared to Cf-penetratin on both of the cell lines examined. Moreover, almost all of the Dabcyl-modified peptides were superior to Cf-Pen except the Dabcyl-Pen(3Nal)(Cf) and the Dabcyl-Pen12(1Nal)(Cf) 2b peptides, and the former derivative had decreased uptake than its acetylated counterpart, although this can be the consequence of FRET phenomena between Dabcyl and Cf. The relatively low cellular uptake of the Dabcyl-Pen12(1Nal)(Cf) 2b peptide compared to Dabcyl-Pen12(Cf) points to the importance of the Trp48 residue in the cellular uptake of dodeca-penetratin. The confocal microscopic studies revealed that besides the greater penetration efficiency of Dabcyl penetratin derivatives, these peptides enter the cytoplasm of cells in an increased manner.

## Figures and Tables

**Table 1:** Chemical characterization of peptides.

Number	Abbreviation	Sequence	R_t_	M_calc._	M_meas._
**1**	Cf-Pen	Cf-RQIKIWFQNRRKWKK	15.1	2471.3	2471.3
**2**	Cf-Pen12	Cf-RQIKIWFRKWKK	15.7	2073.1	2073.1
**3**	Cf-Pen(3Nal)	Cf-RQIKI-*Nal-Nal*-QNRRK-*Nal*-KK	15.3	2543.3	2543.6
**4**	Cf-Pen12(3Nal)	Cf-RQIKI-*Nal*-*Nal*-RK-*Nal*-KK	16.3	2145.1	2145.3
**5**	Cf-Pen(3TIC)	Cf-RQIKI-*TIC*-*TIC*-QNRRK*-TIC*-KK	13.9	2429.3	2429.5
**6**	Cf-Pen12(3TIC)	Cf-RQIKI-*TIC*-*TIC*-RK-*TIC*-KK	14.2	2031.1	2031.2
**7**	Ac-Pen-K(Cf)	Acetyl-RQIKIWFQNRRKWKK-K(Cf)	14.6	2641.4	2641.6
**8**	Dabcyl-Pen-K(Cf)	Dabcyl-RQIKIWFQNRRKWKK-K(Cf)	16.9	2850.5	2850.4
**9**	Ac-Pen12-K(Cf)	Acetyl-RQIKIWFRKWKK-K(Cf)	15.4	2243.2	2243.2
**10**	Dabcyl-Pen12-K(Cf)	Dabcyl-RQIKIWFRKWKK-K(Cf)	18.1	2452.3	2452.3
**11**	Ac-Pen12(Cf)	Acetyl-RQIKIWFRKWKK(Cf)	15.4	2115.1	2114.7
**12**	Dabcyl-Pen12(Cf)	Dabcyl-RQIKIWFRKWKK(Cf)	16.3	2324.2	2323.7
**13**	Ac-Pen12(3Nal)(Cf)	Acetyl-RQIKI-*Nal*-*Nal*-RK-*Nal*-KK(Cf)	17.2	2187.1	2186.7
**14**	Dabcyl-Pen12(3Nal)(Cf)	Dabcyl-RQIKI-*Nal*-*Nal*-RK-*Nal*-KK(Cf)	19.7	2396.2	2395.7
**15**	Ac-Pen12(1Nal)(Cf) 1a,	Acetyl-RQIKIWFRK-*Nal*-KK(Cf)	16.8	2126.1	2126.1
**16**	Dabcyl-Pen12(1Nal)(Cf) 1b,	Dabcyl-RQIKIWFRK-*Nal*-KK(Cf)	18.7	2335.2	2335.2
**17**	Ac-Pen12(1Nal)(Cf) 2a,	Acetyl-RQIKI-*Nal*-FRKWKK(Cf)	17.5	2126.1	2126.1
**18**	Dabcyl-Pen12(1Nal)(Cf) 2b,	Dabcyl-RQIKI-*Nal*-FRKWKK(Cf)	19.9	2335.2	2335.3

Analytical RP-HPLC was performed on Hypersil Hypurity C18 column (4.6 mm × 150 mm, 5 µm, 190 Å). The applied linear gradient elution was 0 min 0% B, 2 min 0% B and 22 min 90% B at a 1 mL/min flow rate. The detection was conducted at λ = 220 nm. The mass spectrometric analysis was performed on a Bruker Amazon SL (Bremen, Germany). The samples were dissolved in acetonitrile–water (50:50, *v*/*v*), containing 0.1% formic acid. Due to the variable temperature while recording the chromatograms, the relations of retention times are not always in accordance with the relations of the hydrophobic character of peptides.

## Data Availability

The original contributions presented in this study are included in the article/Supplementary Materials. Further inquiries can be directed to the corresponding author.

## Supplementary Materials

The following supporting information can be downloaded at: https://www.mdpi.com/article/10.3390/pharmaceutics18050555/s1.
