# Supplementary material for: Penetratin an Old Player in the Field of Cell-Penetrating Peptides Is in New Custom—Effect of Aromatic Non-Natural Amino Acid Substitutions
Source: Pharmaceutics. 2026 Apr 30;18(5):555. doi: 10.3390/pharmaceutics18050555 (PMC13209992; doi:10.3390/pharmaceutics18050555)
Supplement: Supplementary file 1 [file pharmaceutics-18-00555-s001.zip › pharmaceutics-4186662-supplementary.pdf]

Penetratin an Old Player in the Field of Cell-Penetrating Peptides Is in New Custom—Effect of Aromatic Non-Natural Amino Acid Substitutions

Dóra Soltész [1,2], Ildikó Szabó [3], Viktor Farkas [4], Nikolett Borók [1], Tamás Visnovitz [5,6], Dorina Lenzinger [5], Fülöp Károly Grébecz [5], Szilvia Bősze [3,5] and Zoltán Bánóczi [1,*]

[1] Department of Organic Chemistry, Institute of Chemistry, Faculty of Science, ELTE Eötvös Loránd University, Pázmány Péter Sétány 1/A, 1117 Budapest, Hungary; soltesz.dora6@gmail.com (D.S.); borokn29@yahoo.com (N.B.)

[2] Hevesy György PhD School of Chemistry, Institute of Chemistry, ELTE Eötvös Loránd University, Pázmány Péter Sétány 1/A, 1117 Budapest, Hungary

[3] HUN-REN-ELTE Research Group of Peptide Chemistry, 1117 Budapest, Hungary; ildiko.szabo@ttk.elte.hu (I.S.); szilvia.bosze@ttk.elte.hu (S.B.)

[4] HUN-REN-ELTE Protein Modeling Research Group, 1117 Budapest, Hungary; farkas.viktor@ttk.elte.hu

[5] Department of Genetics, Cell- and Immunobiology, Faculty of Medicine, Semmelweis University, Nagyvárad tér 4, 1089 Budapest, Hungary; visnovitz.tamas@semmelweis.hu (T.V.); lenzinger.dorina@semmelweis.hu (D.L.); grebecz.fulop.karoly@semmelweis.hu (F.K.G.)

[6] Department of Plant Physiology and Molecular Plant Biology, ELTE Eötvös Loránd University, Pázmány Péter Sétány 1/c, 1117 Budapest, Hungary

* Correspondence: zoltan.banoczi@ttk.elte.hu

1. Materials

All amino acid derivatives, Fmoc-protected amino acids, N,N'-diisopropylcarbodiimide (DIC), and Rink-amide MBHA resin were purchased from IRIS BiotechGmbH (Marktredwitz, Germany). N,N-diisopropylethylamine (DIEA), 1.8-diazabicyclo[5.4.0]undec-7-ene (DBU), thioanisole, 1,2-ethandithiol (EDT) were obtained from FLUKA (Buchs, Switzerland). Solvents for synthesis and purification were acquired from Molar Chemicals Ltd (Budapest, Hungary). OxymaPure, phenol, 5(6)-carboxyfluorescein (Cf), trifluoroacetic acid (TFA), 5-(N-ethyl-N-isopropyl)amiloride (EIPA), colchicine (COL), sodium-azide (NaN_3), 2-deoxy-D-glucose (DOG) were purchased from Sigma Aldrich (Hungary). Whereas chlorpromazine (CPZ) and methyl-beta-cyclodextrin (CyD) were ordered from TCI chemicals. Dabcyl was acquired from AAT Bioquest.

For the *in vitro* assays DMEM medium, phosphate buffered saline (PBS) trypan blue, and L-glutamine were from Lonza (Basel, Switzerland). Pyruvate, trypsin, paraformaldehyde (PFA), Mowiol 4–88 were obtained from Sigma-Aldrich (Budapest, Hungary). Fetal bovine serum (FBS) and Penicillin/Streptomycin (10,000 units penicillin and 10 mg streptomycin/mL) were from Gibco (Thermo Fisher Scientific, Waltham, MA, USA). HPMI buffer was prepared in our laboratory using components (glucose, $NaHCO_3$, NaCl, N-(2-hydroxyethyl)piperazine-N′-(2-ethanesulfonic acid - HEPES, KCl, $MgCl_2$, $CaCl_2$, $Na_2HPO_4 \times 2\ H_2O$) obtained from Sigma-Aldrich [43]. Hoechst 33342 (62249), LysoTracker Deep Red (L12492, for fixed cells) were from Invitrogen Biotechnology (Thermo Fisher Scientific, Waltham, MA, USA).

1.1 RP-HPLC

Analytical RP-HPLC was performed on Exformma (Exformma Technology (ASIA) Co., Ltd, Hong Kong, China) HPLC system. The used column was Hypersil Hypurity C18 column (4.6 mm × 150 mm, 5 μm, 190 Å). Linear gradient elution (0 min 0% B; 2 min 0% B; 22 min 90% B) was used with eluent A (0.1% TFA in water) and eluent B (0.1% TFA in acetonitrile-water (80:20, v/v)) at 1 mL/min flow rate, and the peaks were detected at λ= 220 nm for both analytical and preparative RP-HPLC. The samples were dissolved in a minimum amount of eluent B and injected into the analytical RP-HPLC. The crude

products were purified on a semi-preparative Phenomenex Jupiter C18 column (250 × 10 mm I.D.) with 10 mm silica (300 Å pore size) (Torrance, CA, USA). The flow rate was 4 mL/min, and linear gradient elution was applied. The samples were dissolved in eluent A containing small percentage of eluent B (10–25% depending on the sequence).

1.2 Mass spectrometry

The molecular weight of peptides was determined with ESI-MS either Bruker Daltonics Esquire 3000 plus (Germany) ion trap mass spectrometer or Bruker Amazon SL (Germany). The samples were dissolved in water-acetonitrile solution (50:50) with 0.1% formic acid. The samples were directly injected with a syringe pump. Parameters: capillary voltage: 4 kV, nebulizer gas: 10 psi, dry gas:4 L/min, heated capillary temperature: 250 °C.

1.3 Cell Culture

EBC-1 human lung squamous cancer cells (CRL-5889™) were a generous gift of Prof. László Kőhidai from Semmelweis University, Faculty of Medicine, Department of Genetics, Cell- and Immunobiology. A-431 human epidermoid cancer cells were generous gift from Prof József Tóvári, National Institute of Oncology The cell lines maintained in Dulbecco's Modified Eagle Medium (DMEM) supplemented with 10% heat inactivated foetal calf serum (FCS), L-Glutamine (2 mM), 1% nonessential amino acids and 1% penicillin-streptomycin (from 10,000 units penicillin and 10mg/ml streptomycin). Cells were maintained in sterile T25 and T75 flasks with ventilation cap (Sarstedt, Nümbrecht, Germany) at 37 °C in a humidified atmosphere with 5% CO2 in ESCO Cell Culture Incubator (ESCO, Friedberg, Germany). Manipulations with the cells were performed in the laminar biosafety cabinet ESCO Sentinel Gold class II model AC2-4E8 (ESCO). No mycoplasma contamination was detected in the cell cultures.

Prior to flow cytometry analysis, trypsin enzyme was used to detach the adherent cells from the plate and and also to remove membrane-bound peptides.

1.4 Determination of in vitro cellular internalization

The intracellular fluorescence intensity of cells was measured at λex = 488 nm (Coherent Sapphire laser excitation, emission channel—LP 510, BP 530/30). The results were analyzed with FACSDiva software. All measurements were performed in triplicates.

2. Chemical characterization of peptide conjugates

2.1. RP-HPLC

Analytical RP-HPLC was measured on Exformma (Exformma Technology (ASIA) Co., Ltd, Hong Kong, China) HPLC system. The peptide conjugates were injected on Hypersil Hypurity C18 column (4.6 mm × 150 mm, 5 µm, 190 Å). Linear gradient elution (0 min 0% B; 2 min 0% B; 22 min 90% B) was used with eluent A (0.1% TFA in water) and eluent B (0.1% TFA in acetonitrile-water (80:20, *v/v*)) at 1 mL/min flow rate, the peaks were detected at λ= 220 nm for both analytical and preparative RP-HPLC. The samples were dissolved in a minimum amount of eluent B and injected into the analytical RP-HPLC. The crude products were purified on a semi-preparative Phenomenex Jupiter C18 column (250 × 10 mm I.D.) with 10 mm silica (300 Å pore size) (Torrance, CA, USA). Flow rate was 4 mL/min and linear gradient elution was applied. The samples were dissolved in eluent A containing small percentage of eluent B (10–25% depending on sequence).

In some cases two peaks can be observed on the chromatogram- these belong to the same peptide coupled with either 5- or 6- carboxyfluorescein. The isomers co-eluted on the semi-preparative HPLC column, thereby we could not separate them.

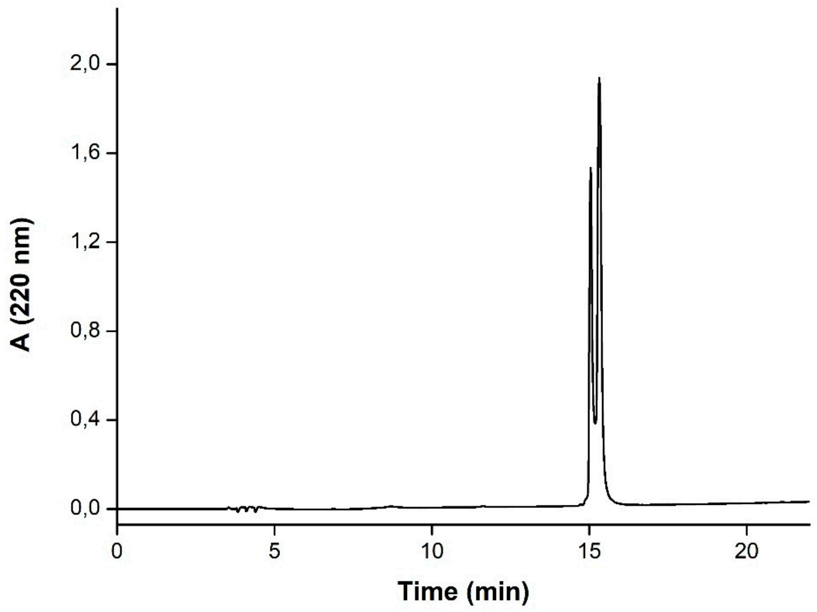

Figure S1. HPLC chromatogram of Cf-Pen. Retention time was obtained on Hypersil Hypurity C18 column (4.6 mm x 150 mm, 5 μm, 190 Å). The applied linear gradient elution was 0 min 0% B, 2 min 0% B, 22 min 90% B at 1 mL/min flow rate. The detection was carried on at λ = 220 nm.

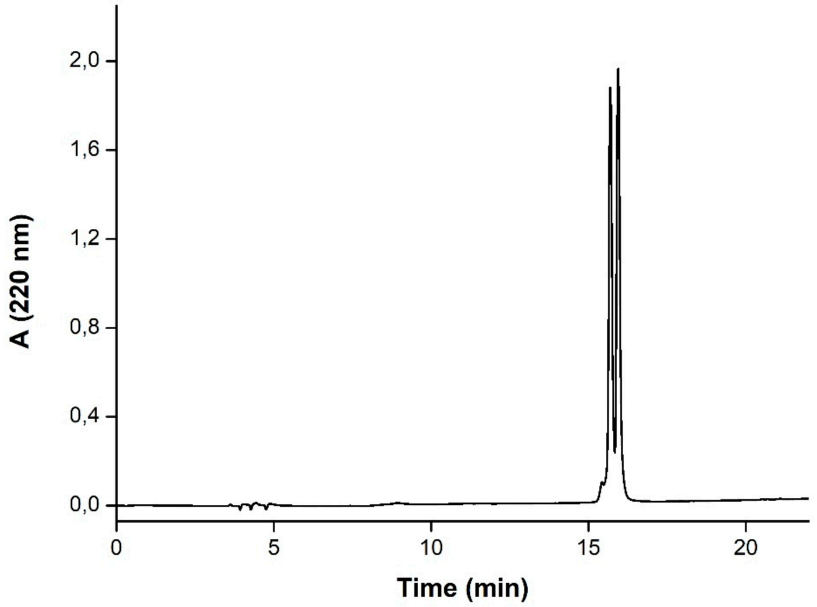

Figure S2. HPLC chromatogram of Cf-Pen12. Retention time was obtained on Hypersil Hypurity C18 column (4.6 mm x 150 mm, 5 μm, 190 Å). The applied linear gradient elution was 0 min 0% B, 2 min 0% B, 22 min 90% B at 1 mL/min flow rate. The detection was carried on at λ = 220 nm.

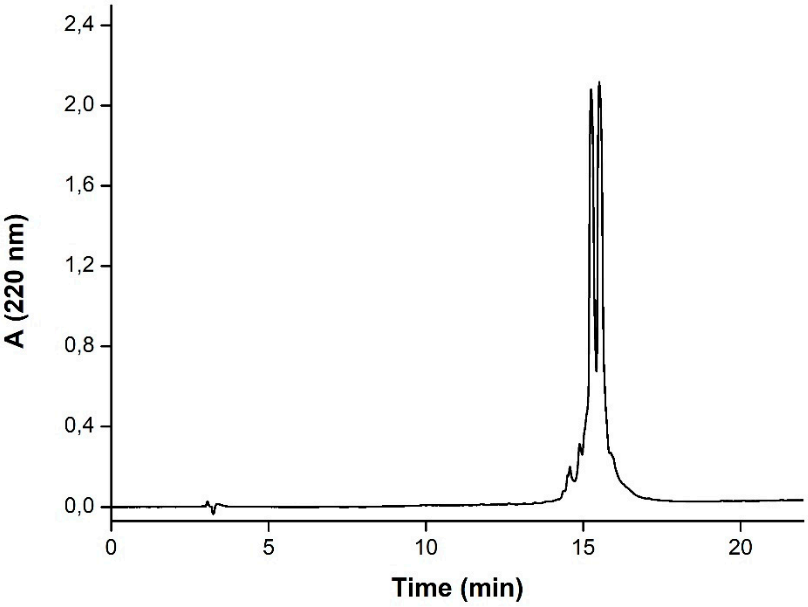

Figure S3. HPLC chromatogram of Cf-Pen(3Nal). Retention time was obtained on Hypersil Hypurity C18 column (4.6 mm x 150 mm, 5 µm, 190 Å). The applied linear gradient elution was 0 min 0% B, 2 min 0% B, 22 min 90% B at 1 mL/min flow rate. The detection was carried on at λ = 220 nm.

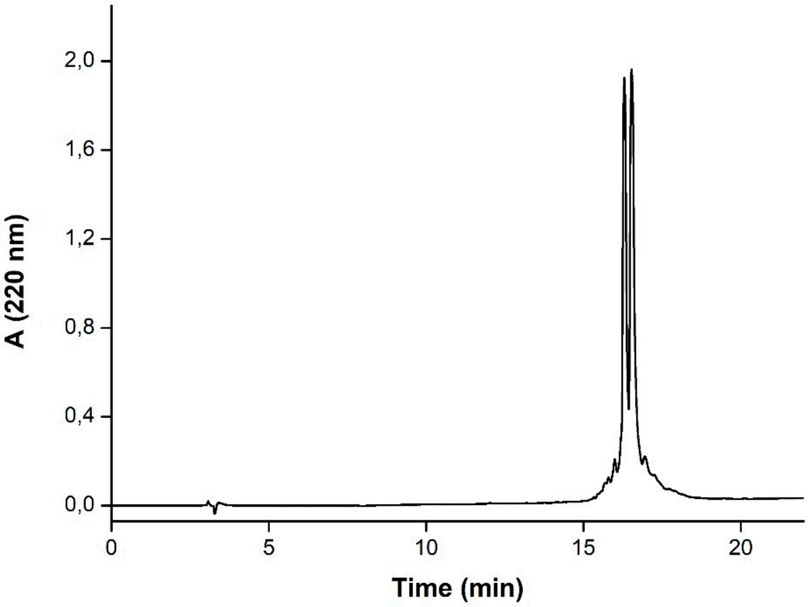

Figure S4. HPLC chromatogram of Cf-Pen12(3Nal). Retention time was obtained on Hypersil Hypurity C18 column (4.6 mm x 150 mm, 5 µm, 190 Å). The applied linear gradient elution was 0 min 0% B, 2 min 0% B, 22 min 90% B at 1 mL/min flow rate. The detection was carried on at λ = 220 nm.

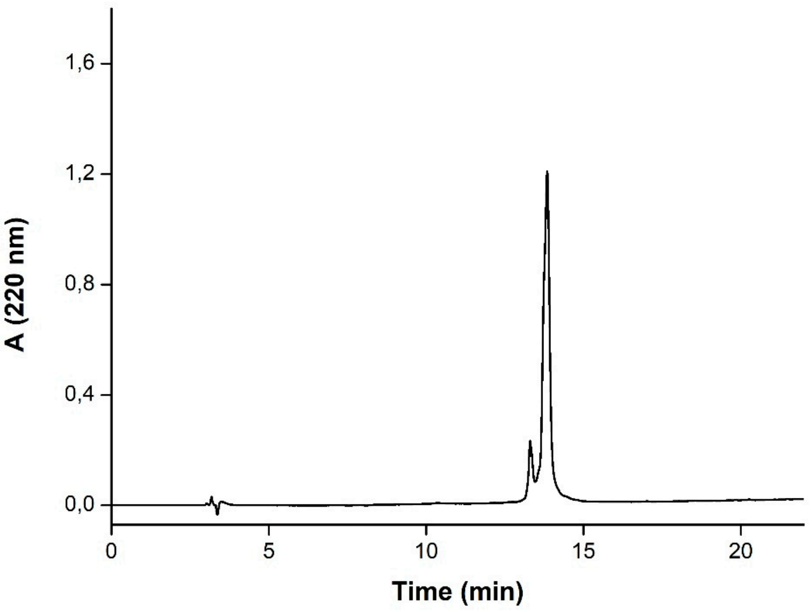

Figure S5. HPLC chromatogram of Cf-Pen(3Tic). Retention time was obtained on Hypersil Hypurity C18 column (4.6 mm x 150 mm, 5 μm, 190 Å). The applied linear gradient elution was 0 min 0% B, 2 min 0% B, 22 min 90% B at 1 mL/min flow rate. The detection was carried on at λ = 220 nm.

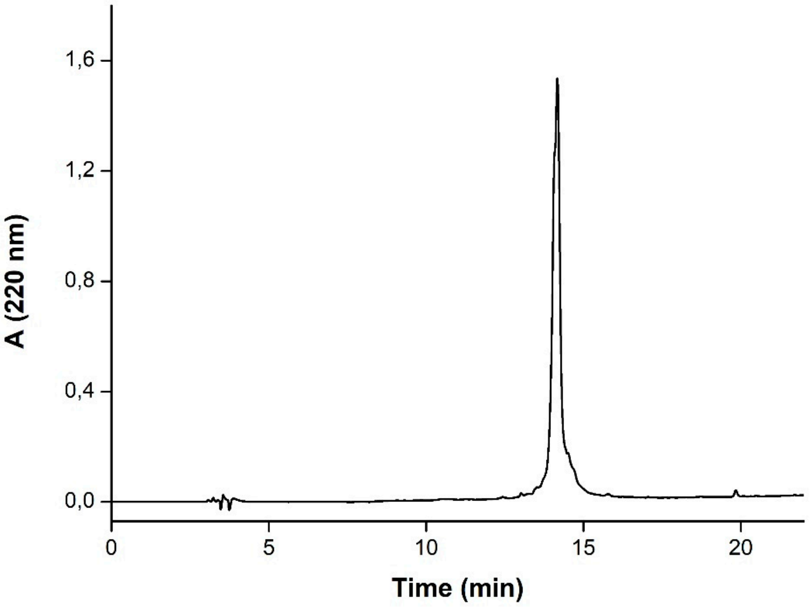

Figure S6. HPLC chromatogram of Cf-Pen12(3Tic). Retention time was obtained on Hypersil Hypurity C18 column (4.6 mm x 150 mm, 5 μm, 190 Å). The applied linear gradient elution was 0 min 0% B, 2 min 0% B, 22 min 90% B at 1 mL/min flow rate. The detection was carried on at λ = 220 nm.

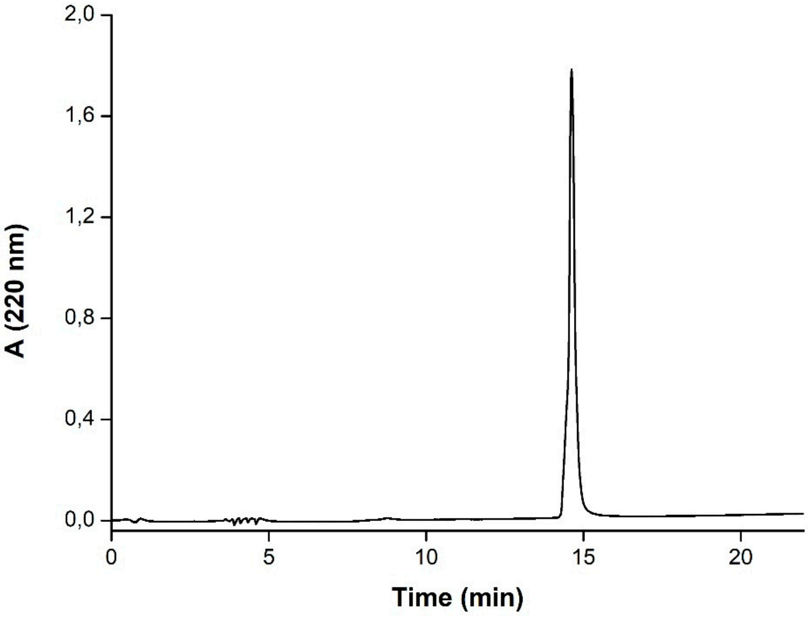

Figure S7. HPLC chromatogram of Ac-Pen-Lys(Cf). Retention time was obtained on Hypersil Hypurity C18 column (4.6 mm x 150 mm, 5 μm, 190 Å). The applied linear gradient elution was 0 min 0% B, 2 min 0% B, 22 min 90% B at 1 mL/min flow rate. The detection was carried on at λ = 220 nm.

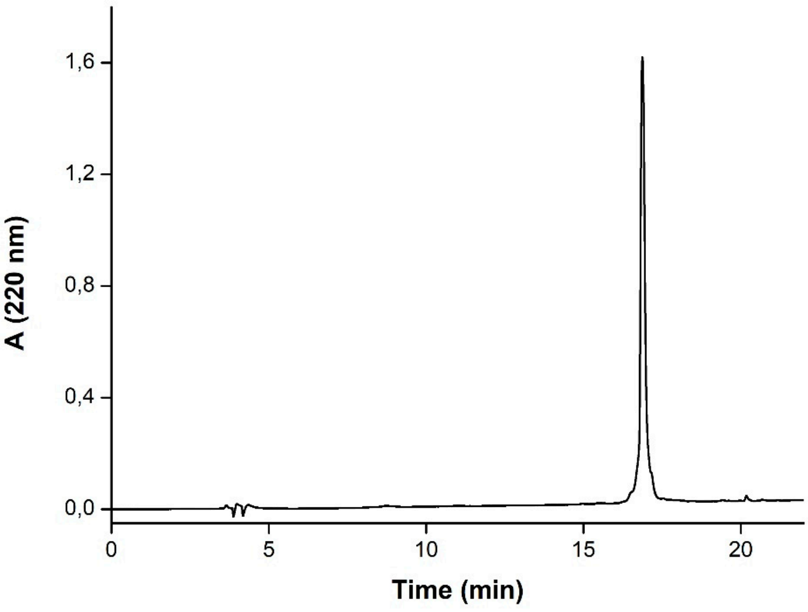

Figure S8. HPLC chromatogram of Dabcyl-Pen-Lys(Cf). Retention time was obtained Hypersil Hypurity C18 column (4.6 mm x 150 mm, 5 μm, 190 Å). The applied linear gradient elution was 0 min 0% B, 2 min 0% B, 22 min 90% B at 1 mL/min flow rate. The detection was carried on at λ = 220 nm.

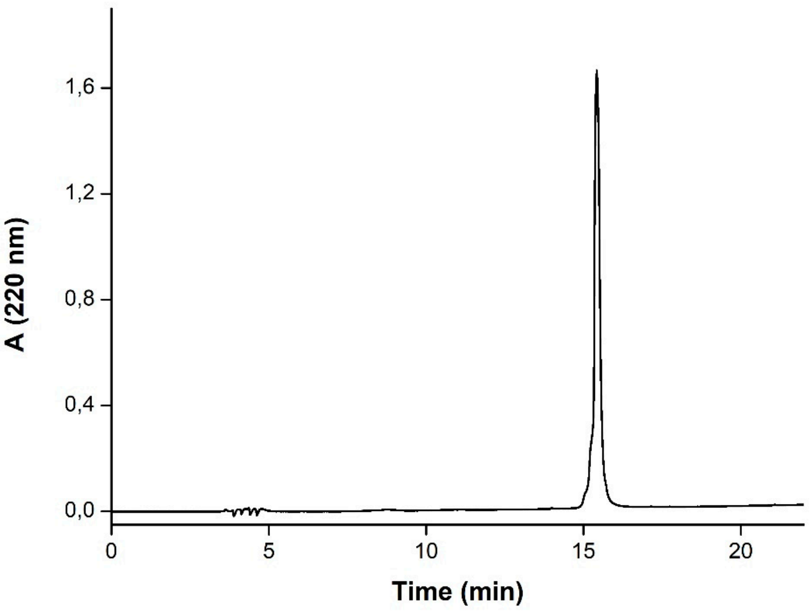

Figure S9. HPLC chromatogram of Ac-Pen12-Lys(Cf). Retention time was obtained on obtained Hypersil Hypurity C18 column (4.6 mm x 150 mm, 5 μm, 190 Å). The applied linear gradient elution was 0 min 0% B, 2 min 0% B, 22 min 90% B at 1 mL/min flow rate. The detection was carried on at λ = 220 nm.

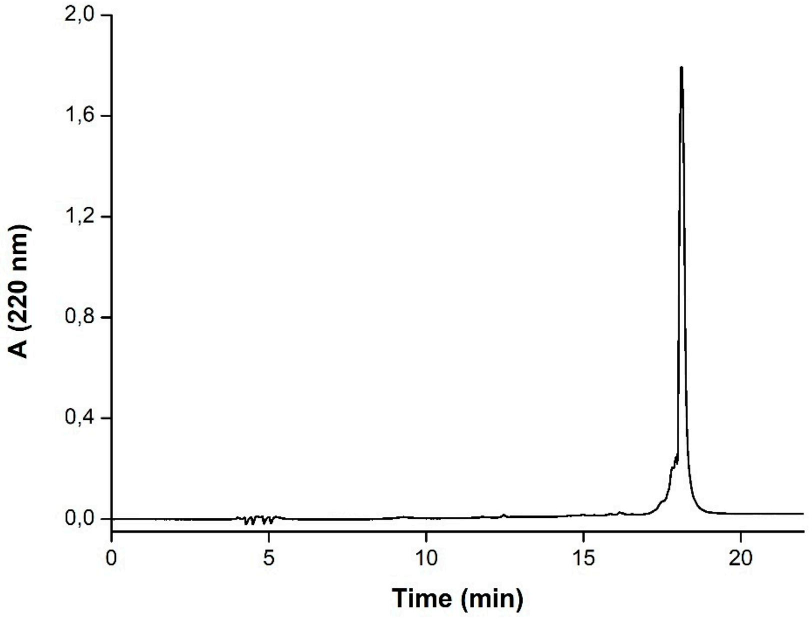

Figure S10. HPLC chromatogram of Dabcyl-Pen12-Lys(Cf). Retention time was obtained on Hypersil Hypurity C18 column (4.6 mm x 150 mm, 5 μm, 190 Å). The applied linear gradient elution was 0 min 0% B, 2 min 0% B, 22 min 90% B at 1 mL/min flow rate. The detection was carried on at λ = 220 nm.

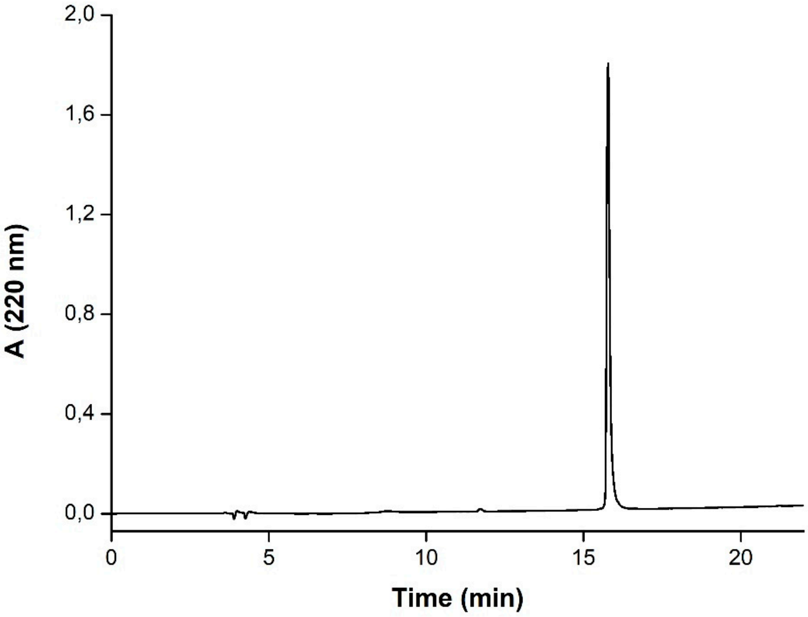

Figure S11. HPLC chromatogram of Ac-Pen12(Cf). Retention time was obtained on Hypersil Hypurity C18 column (4.6 mm x 150 mm, 5 µm, 190 Å). The applied linear gradient elution was 0 min 0% B, 2 min 0% B, 22 min 90% B at 1 mL/min flow rate. The detection was carried on at λ = 220 nm.

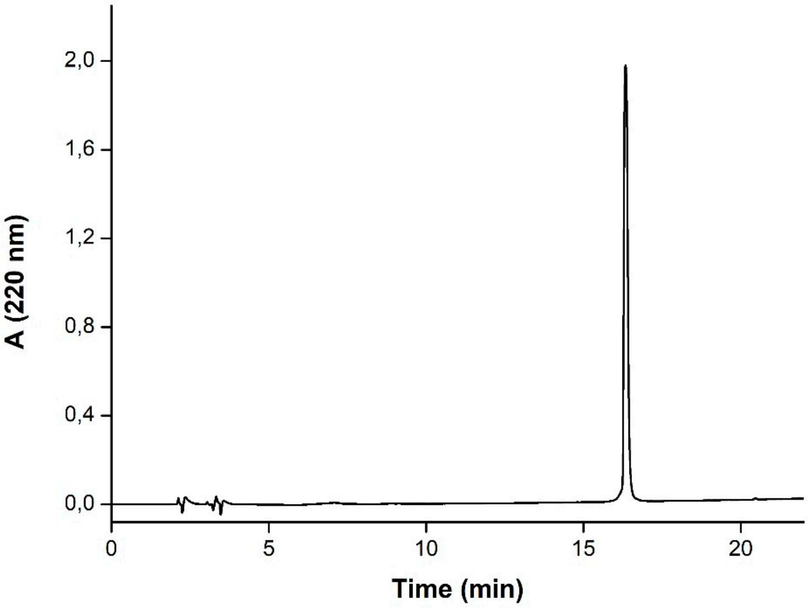

Figure S12. HPLC chromatogram of Dabcyl-Pen12(Cf). Retention time was obtained on Hypersil Hypurity C18 column (4.6 mm x 150 mm, 5 µm, 190 Å). The applied linear gradient elution was 0 min 0% B, 2 min 0% B, 22 min 90% B at 1 mL/min flow rate. The detection was carried on at λ = 220 nm.

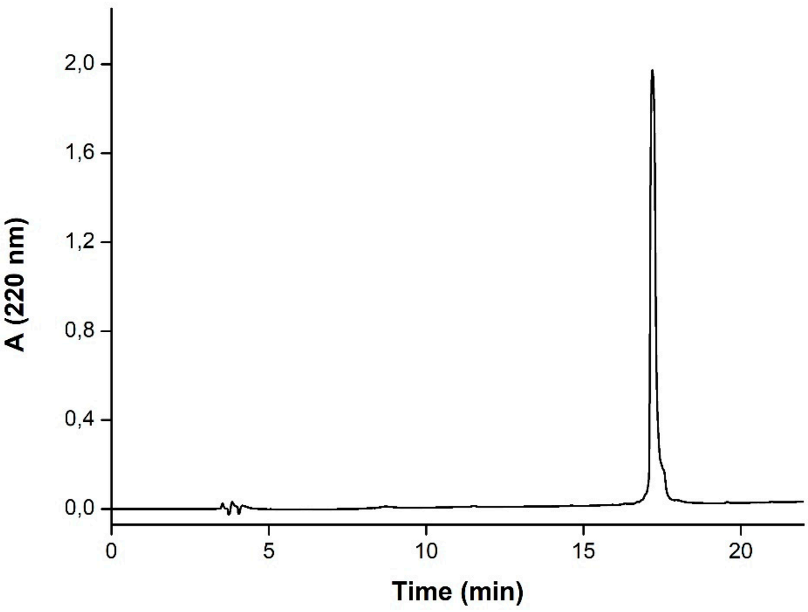

Figure S13. HPLC chromatogram of Ac-Pen12(3Nal)(Cf). Retention time was obtained on Hypersil Hypurity C18 column (4.6 mm x 150 mm, 5 μm, 190 Å). The applied linear gradient elution was 0 min 0% B, 2 min 0% B, 22 min 90% B at 1 mL/min flow rate. The detection was carried on at λ = 220 nm.

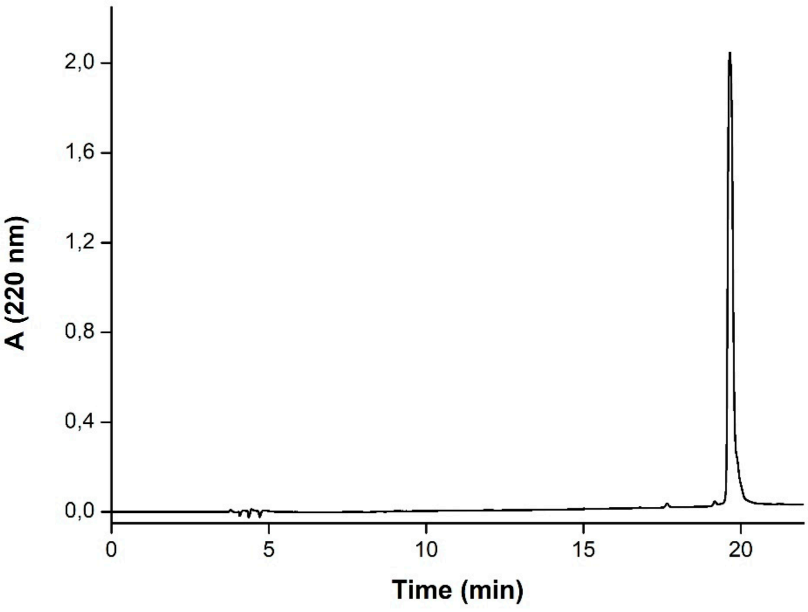

Figure S14. HPLC chromatogram of Dabcyl-Pen12(3Nal)(Cf). Retention time was obtained on Hypersil Hypurity C18 column (4.6 mm x 150 mm, 5 μm, 190 Å). The applied linear gradient elution was 0 min 0% B, 2 min 0% B, 22 min 90% B at 1 mL/min flow rate. The detection was carried on at λ = 220 nm.

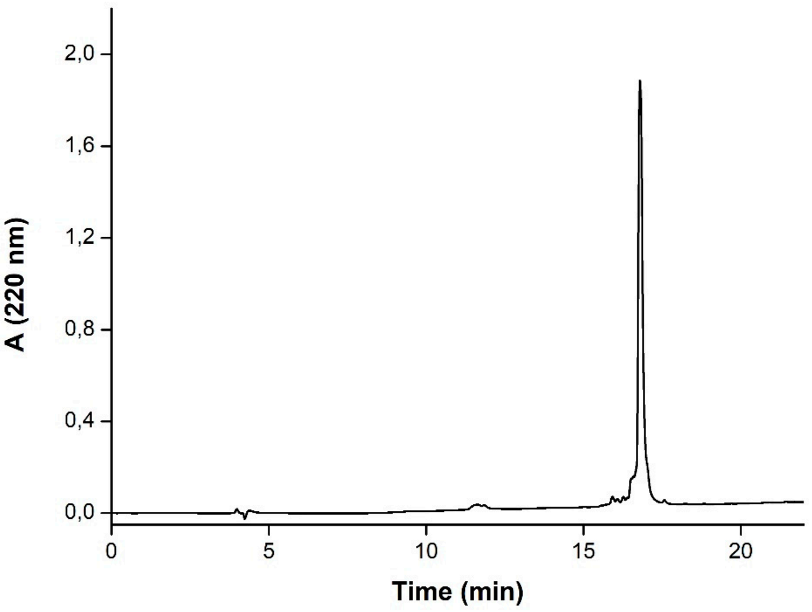

Figure S15. HPLC chromatogram of Ac-Pen12(1Nal)(Cf) 1a. Retention time was obtained on Hypersil Hypurity C18 column (4.6 mm x 150 mm, 5 μm, 190 Å). The applied linear gradient elution was 0 min 0% B, 2 min 0% B, 22 min 90% B at 1 mL/min flow rate. The detection was carried on at λ = 220 nm.

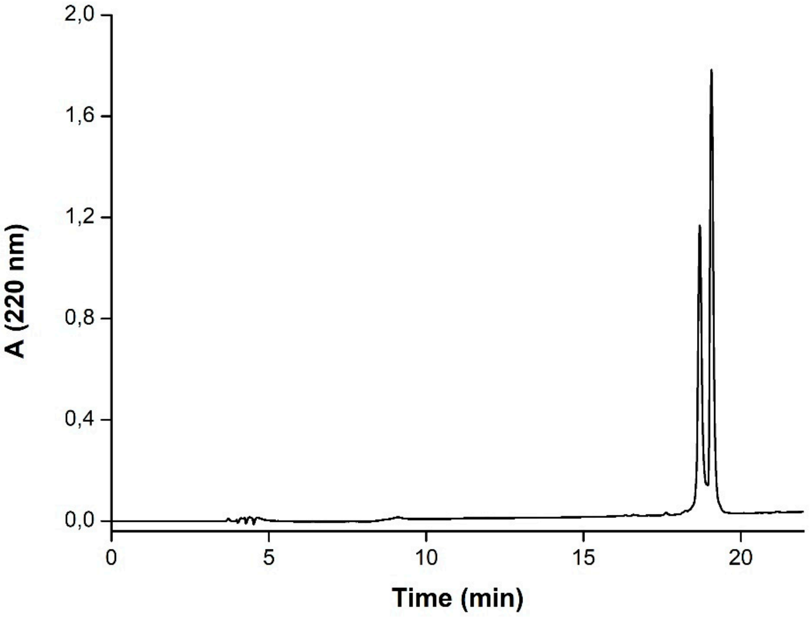

Figure S16. HPLC chromatogram of Dabcyl-Pen12(1Nal)(Cf) 1b. Retention time was obtained on Hypersil Hypurity C18 column (4.6 mm x 150 mm, 5 μm, 190 Å). The applied linear gradient elution was 0 min 0% B, 2 min 0% B, 22 min 90% B at 1 mL/min flow rate. The detection was carried on at λ = 220 nm.

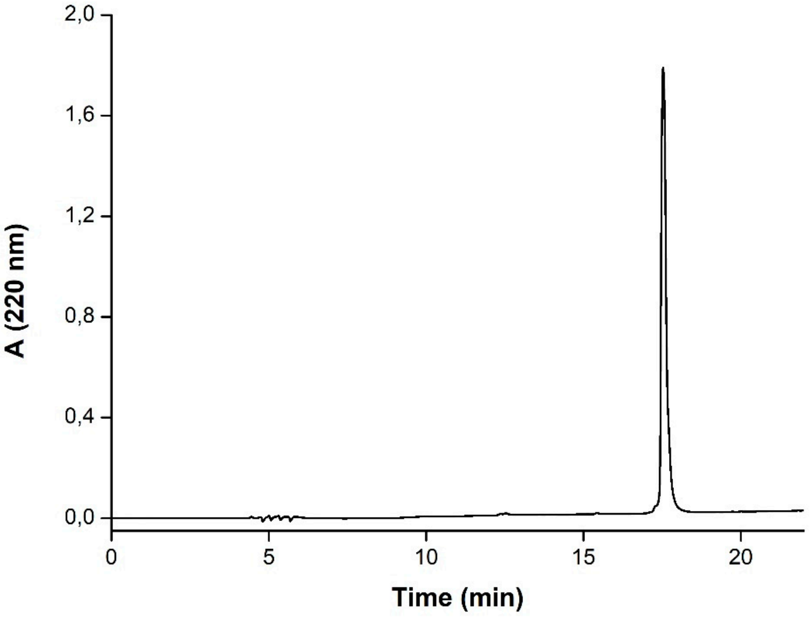

Figure S17. HPLC chromatogram of Ac-Pen12(1Nal)(Cf) 2a. Retention time was obtained on Hypersil Hypurity C18 column (4.6 mm x 150 mm, 5 μm, 190 Å). The applied linear gradient elution was 0 min 0% B, 2 min 0% B, 22 min 90% B at 1 mL/min flow rate. The detection was carried on at λ = 220 nm.

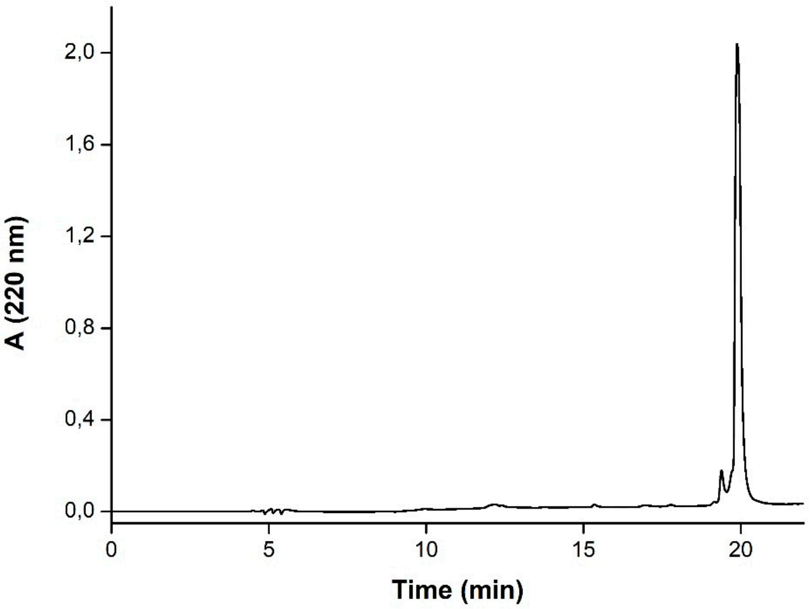

Figure S18. HPLC chromatogram of Dabcyl-Pen12(1Nal)(Cf) 2b. Retention time was obtained on Hypersil Hypurity C18 column (4.6 mm x 150 mm, 5 μm, 190 Å). The applied linear gradient elution was 0 min 0% B, 2 min 0% B, 22 min 90% B at 1 mL/min flow rate. The detection was carried on at λ = 220 nm.

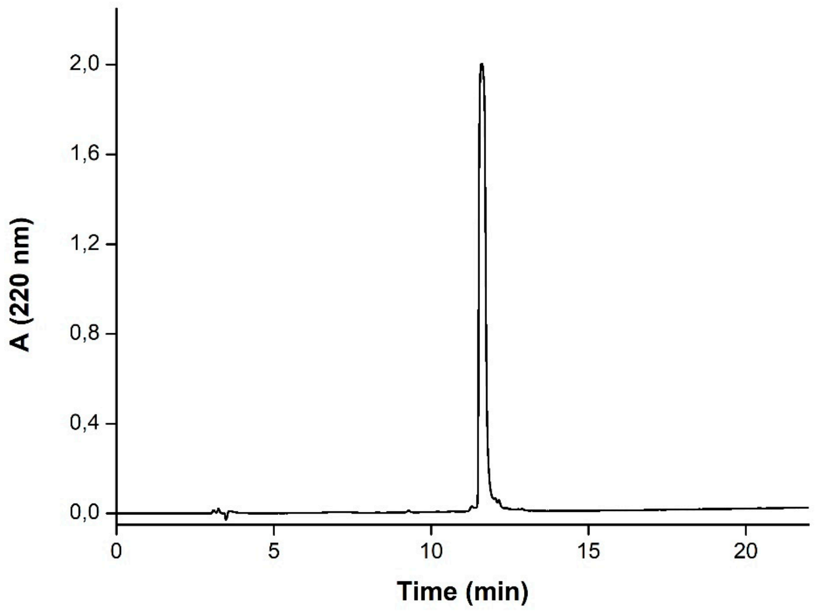

Figure S19. HPLC chromatogram of Cf-(Arg)8. Retention time was obtained on Hypersil Hypurity C18 column (4.6 mm x 150 mm, 5 μm, 190 Å). The applied linear gradient elution was 0 min 0% B, 2 min 0% B, 22 min 90% B at 1 mL/min flow rate. The detection was carried on at λ = 220 nm.

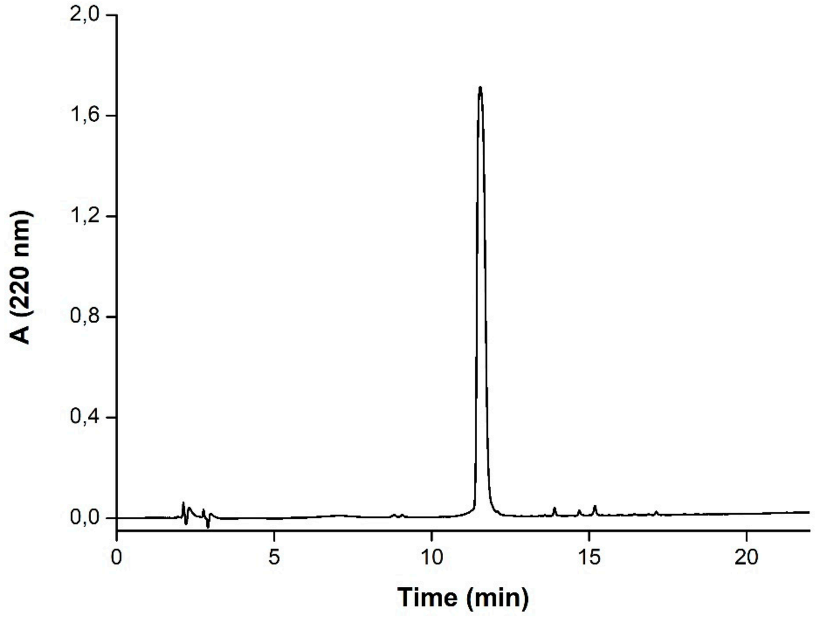

Figure S20. HPLC chromatogram of Pen12. Retention time was obtained on Hypersil Hypurity C18 column (4.6 mm x 150 mm, 5 μm, 190 Å). The applied linear gradient elution was 0 min 0% B, 2 min 0% B, 22 min 90% B at 1 mL/min flow rate. The detection was carried on at λ = 220 nm.

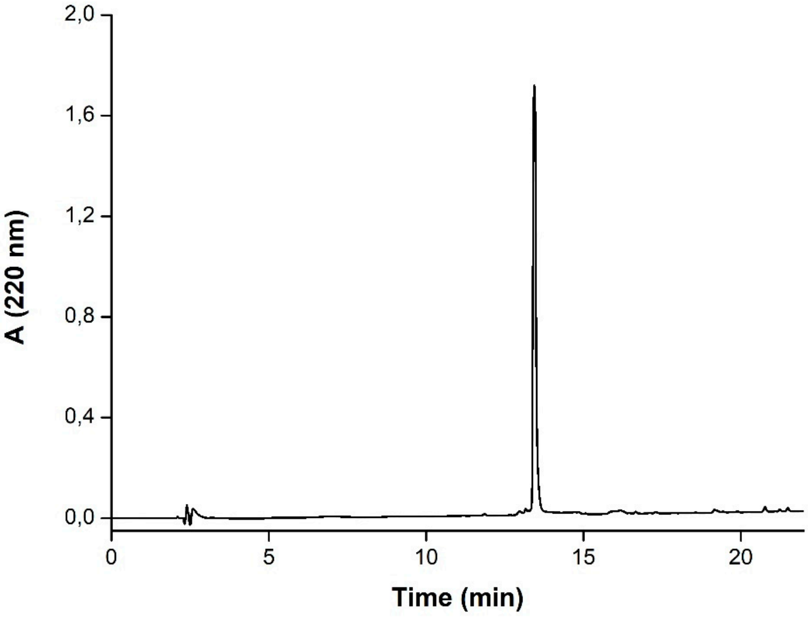

Figure S21. HPLC chromatogram of Pen12(3Nal). Retention time was obtained on Hypersil Hypurity C18 column (4.6 mm x 150 mm, 5 μm, 190 Å). The applied linear gradient elution was 0 min 0% B, 2 min 0% B, 22 min 90% B at 1 mL/min flow rate. The detection was carried on at λ = 220 nm.

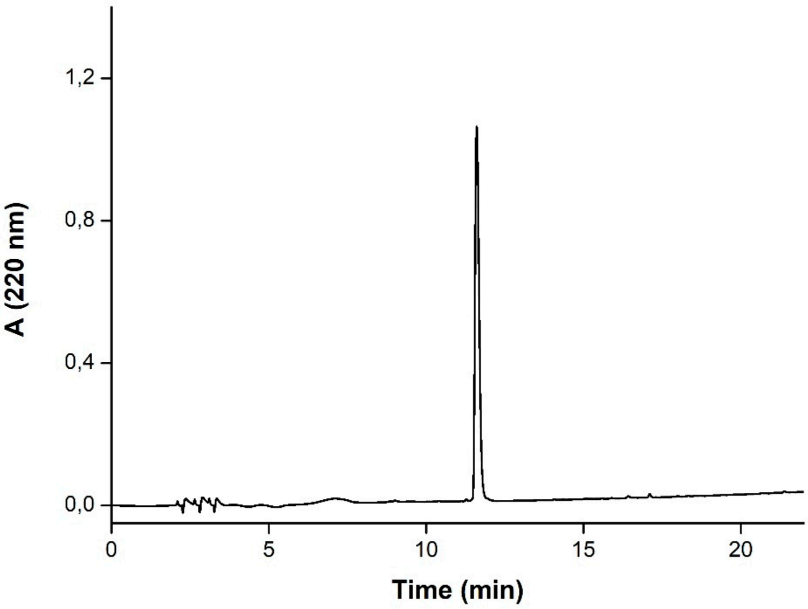

Figure S22. HPLC chromatogram of Pen12(3TIC). Retention time was obtained on Hypersil Hypurity C18 column (4.6 mm x 150 mm, 5 μm, 190 Å). The applied linear gradient elution was 0 min 0% B, 2 min 0% B, 22 min 90% B at 1 mL/min flow rate. The detection was carried on at λ = 220 nm.

2.2 Mass spectrometry

The molecular weight of peptides was determined with ESI-MS using Bruker Amazon SL (Germany) ion-trap mass spectrometer. The samples were dissolved in water-acetonitrile solution (50:50) with 0.1% formic acid. The samples were directly injected with a syringe pump. Parameters: capillary voltage: 4 kV, nebulizer gas: 10 psi, dry gas:4 L/min, heated capillary temperature: 250 °C.

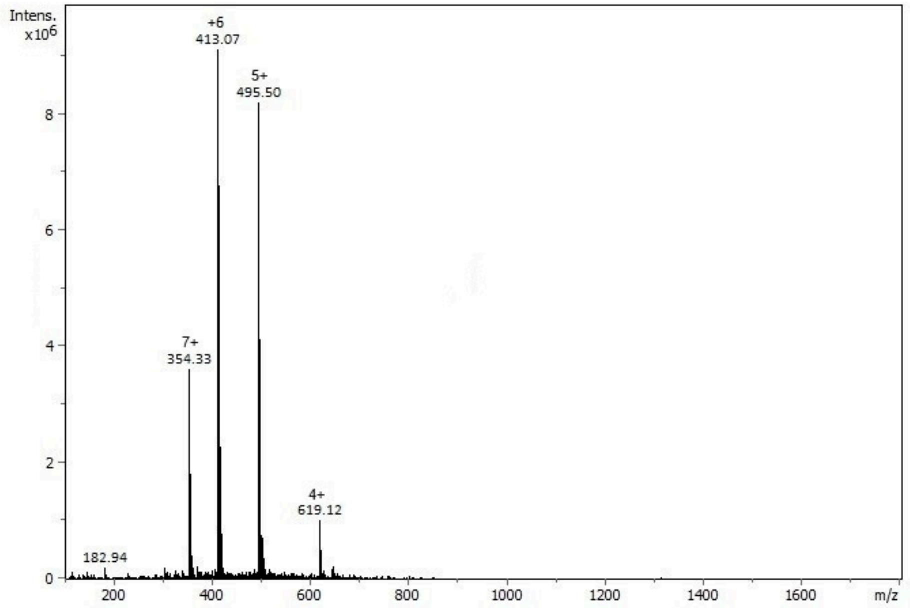

Figure S23. MS Spectrum of Cf-Pen. The identity of the peptide conjugate was determined using Bruker Amazon SL (Germany) ion trap mass spectrometer. The samples are dissolved in water-acetonitrile (50:50) with 0.1% formic acid.

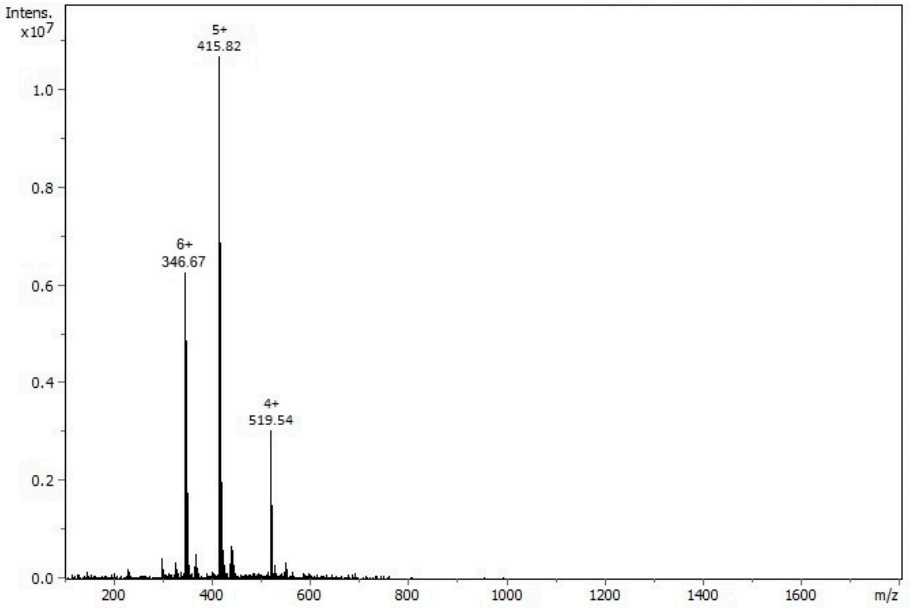

Figure S24. MS Spectrum of Cf-Pen12. The identity of the peptide conjugate was determined using Bruker Amazon SL (Germany) ion trap mass spectrometer. The samples are dissolved in water-acetonitrile (50:50) with 0.1% formic acid.

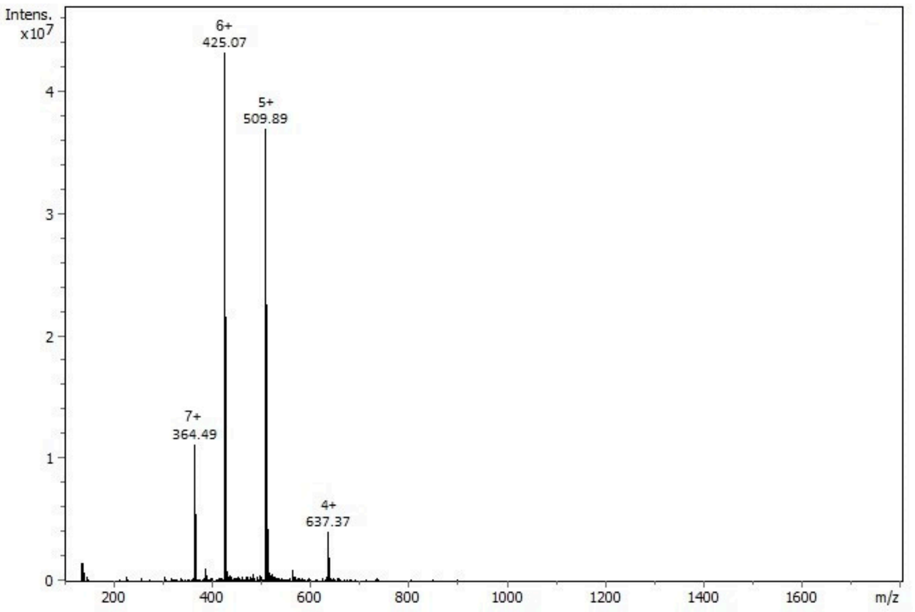

Figure S25. MS Spectrum of Cf-Pen(3Nal). The identity of the peptide conjugate was determined using Bruker Amazon SL (Germany) ion trap mass spectrometer. The samples are dissolved in water-acetonitrile (50:50) with 0.1% formic acid.

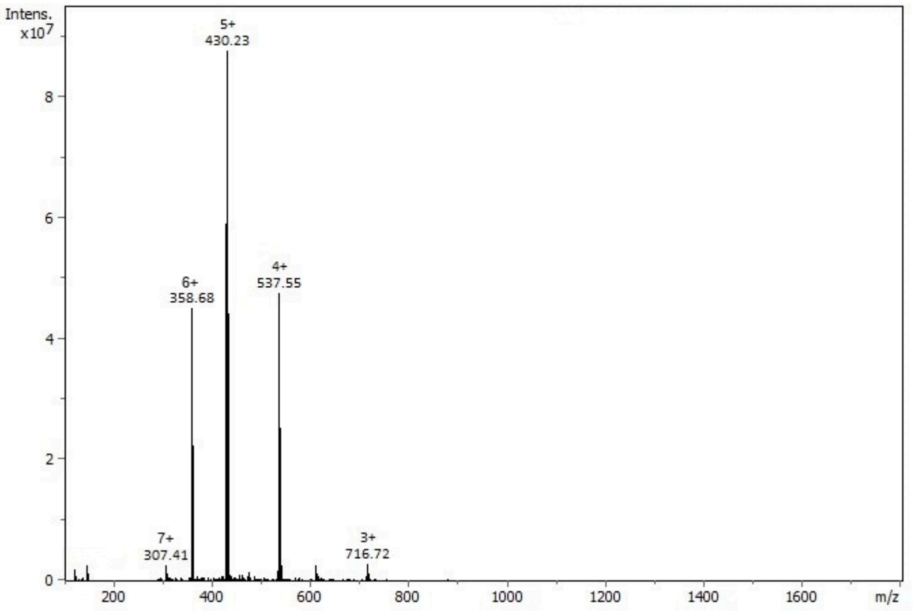

Figure S26. MS Spectrum of Cf-Pen12(3Nal). The identity of the peptide conjugate was determined using Bruker Amazon SL (Germany) ion trap mass spectrometer. The samples are dissolved in water-acetonitrile (50:50) with 0.1% formic acid.

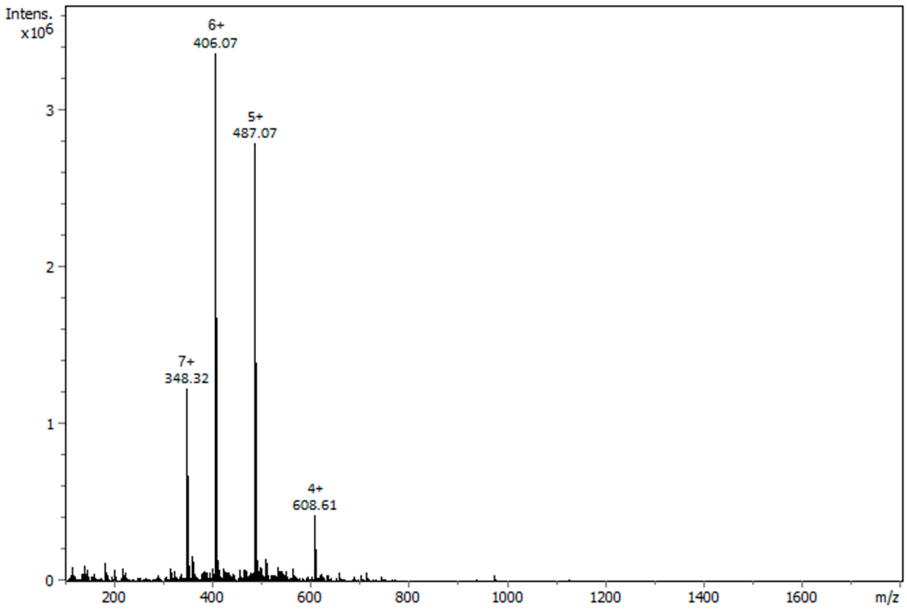

Figure S27. MS Spectrum of Cf-Pen(3Tic). The identity of the peptide conjugate was determined using Bruker Amazon SL (Germany) ion trap mass spectrometer. The samples are dissolved in water-acetonitrile (50:50) with 0.1% formic acid.

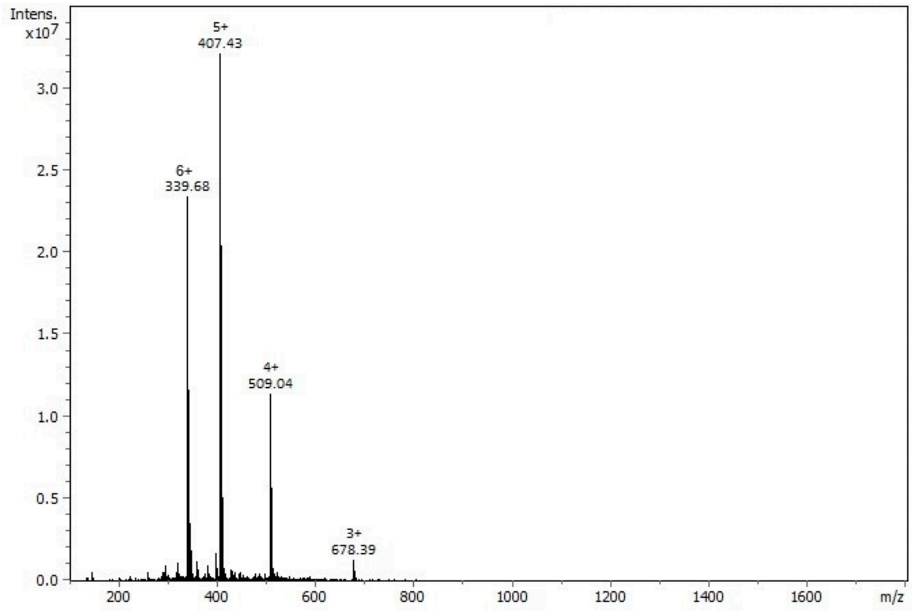

Figure S28. MS Spectrum of Cf-Pen12(3Tic). The identity of the peptide conjugate was determined using Bruker Amazon SL (Germany) ion trap mass spectrometer. The samples are dissolved in water-acetonitrile (50:50) with 0.1% formic acid.

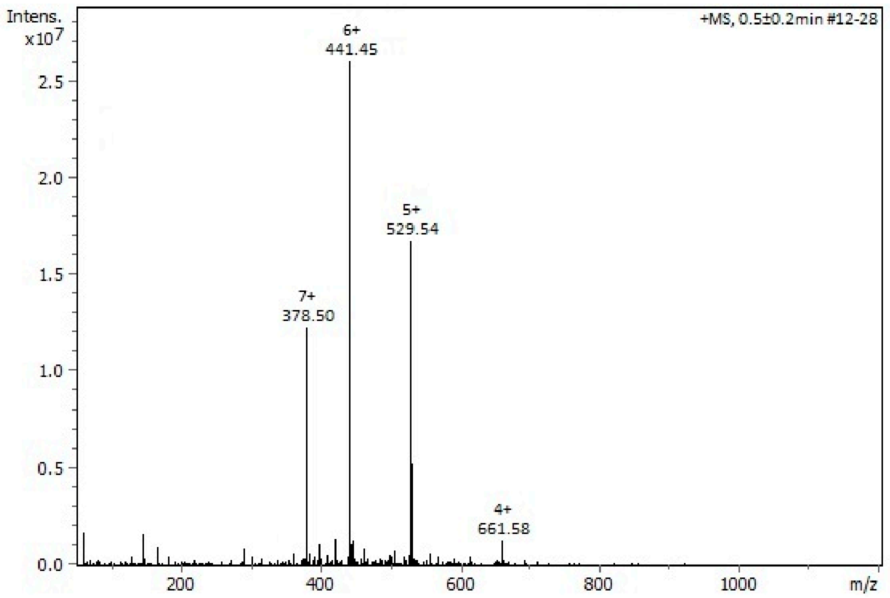

Figure S29. MS Spectrum of Ac-Pen-Lys(Cf). The identity of the peptide conjugate was determined using Bruker Amazon SL (Germany) ion trap mass spectrometer. The samples are dissolved in water-acetonitrile (50:50) with 0.1% formic acid.

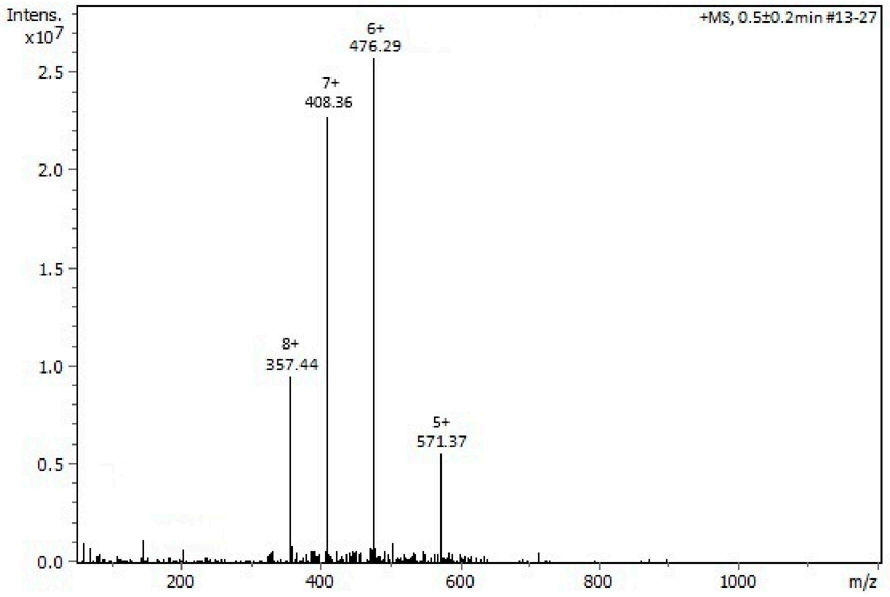

Figure S30. MS Spectrum of of Dabcyl-Pen-Lys(Cf). The identity of the peptide conjugate was determined using Bruker Amazon SL (Germany) ion trap mass spectrometer. The samples are dissolved in water-acetonitrile (50:50) with 0.1% formic acid.

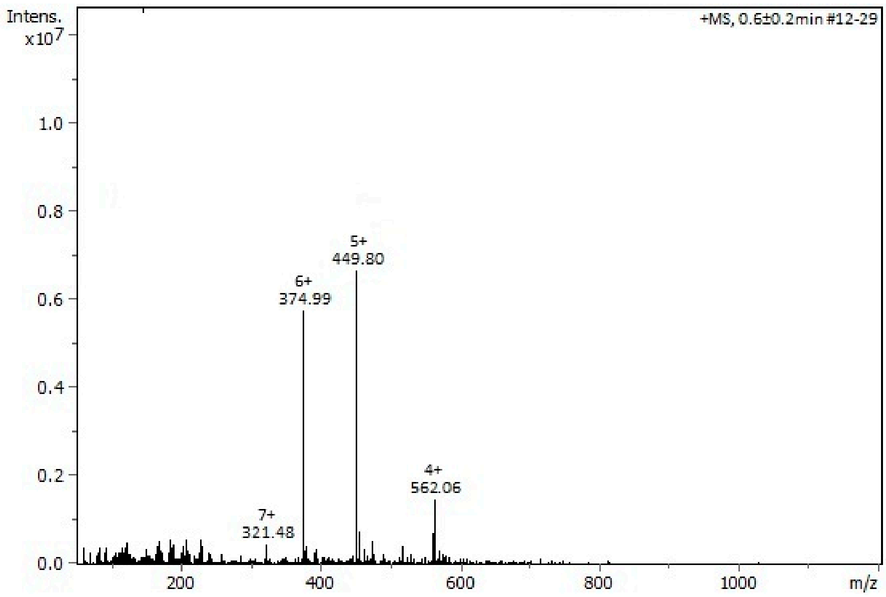

Figure S31. MS Spectrum of Ac-Pen12-Lys(Cf). The identity of the peptide conjugate was determined using Bruker Amazon SL (Germany) ion trap mass spectrometer. The samples are dissolved in water-acetonitrile (50:50) with 0.1% formic acid.

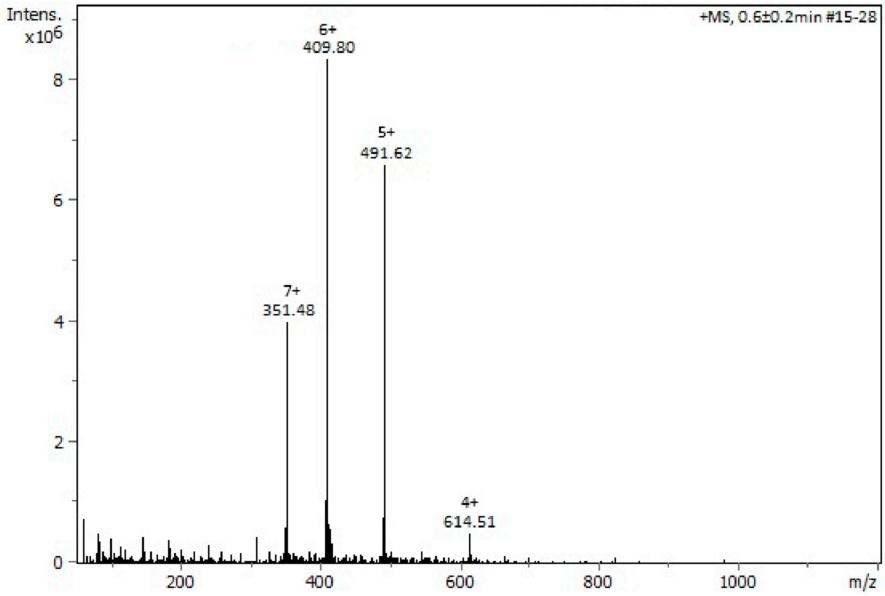

Figure S32. MS Spectrum of Dabcyl-Pen12-Lys(Cf). The identity of the peptide conjugate was determined using Bruker Amazon SL (Germany) ion trap mass spectrometer. The samples are dissolved in water-acetonitrile (50:50) with 0.1% formic acid.

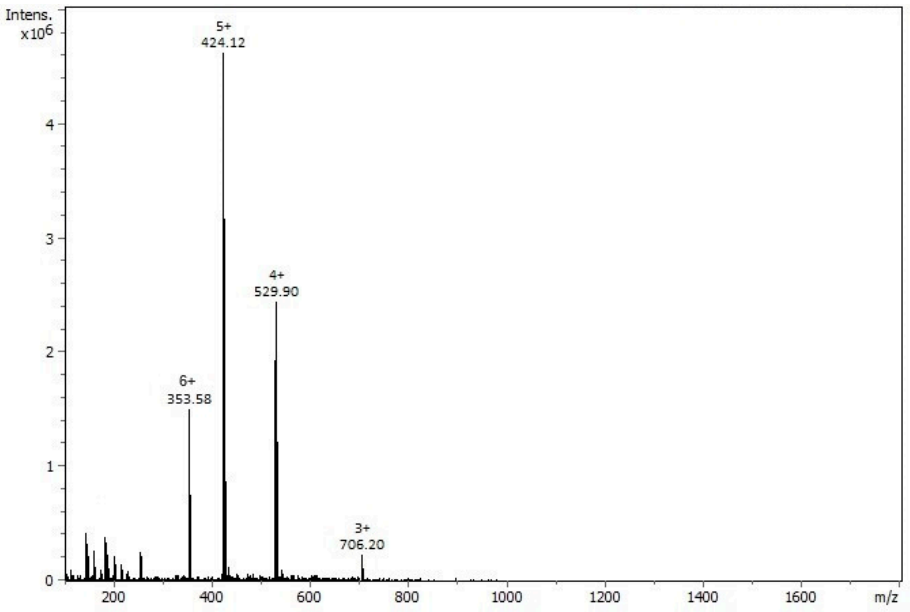

Figure S33. MS Spectrum of Ac-Pen12(Cf). The identity of the peptide conjugate was determined using Bruker Amazon SL (Germany) ion trap mass spectrometer. The samples are dissolved in water-acetonitrile (50:50) with 0.1% formic acid.

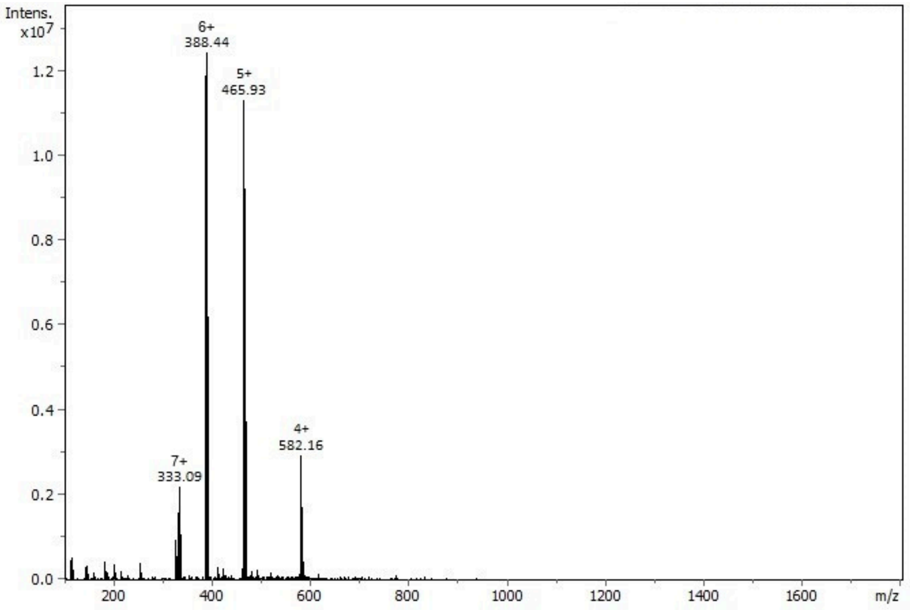

Figure S34. MS Spectrum of Dabcyl-Pen12(Cf). The identity of the peptide conjugate was determined using Bruker Amazon SL (Germany) ion trap mass spectrometer. The samples are dissolved in water-acetonitrile (50:50) with 0.1% formic acid.

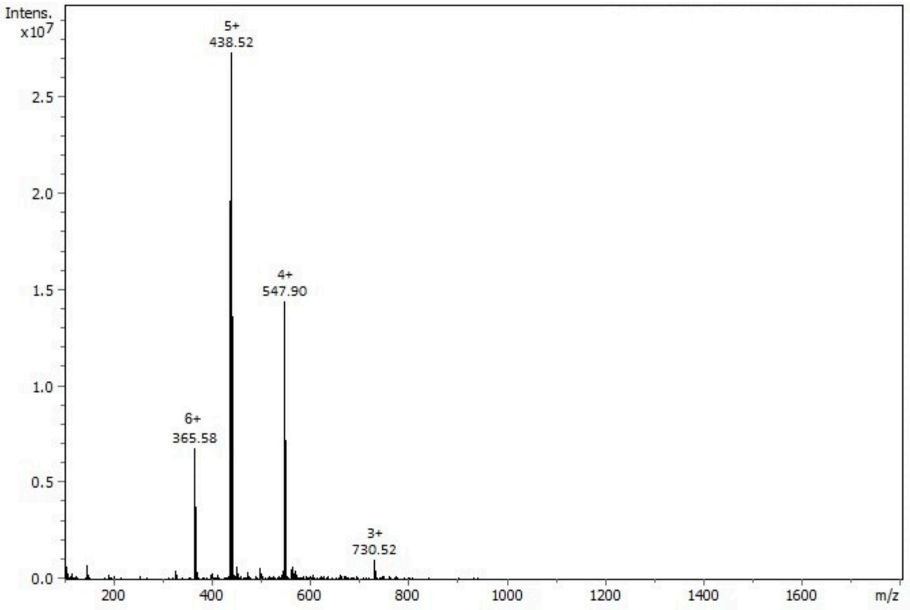

Figure S35. MS Spectrum of Ac-Pen12(3Nal)(Cf). The identity of the peptide conjugate was determined using Bruker Amazon SL (Germany) ion trap mass spectrometer. The samples are dissolved in water-acetonitrile (50:50) with 0.1% formic acid.

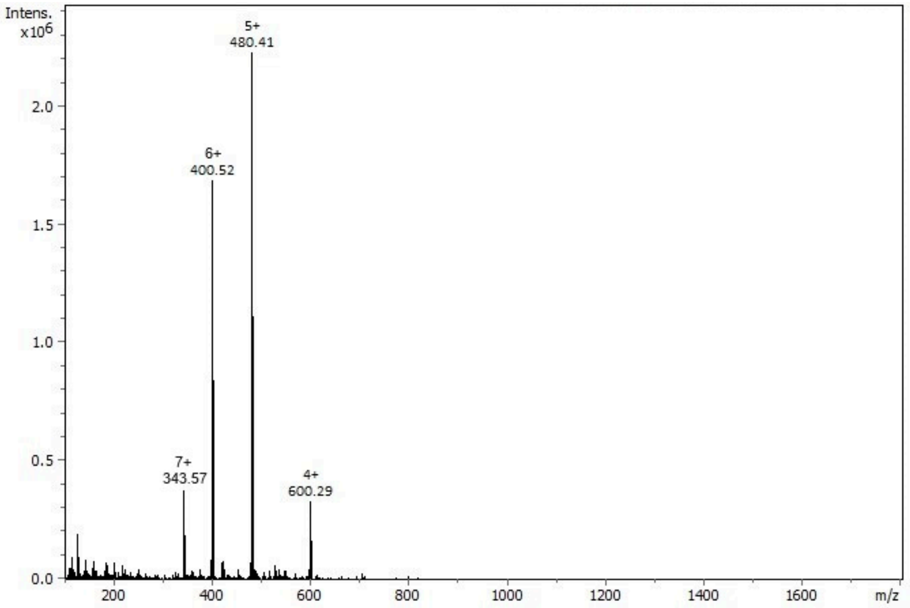

Figure S36. MS Spectrum of Dabcyl-Pen12(3Nal)(Cf). The identity of the peptide conjugate was determined using Bruker Amazon SL (Germany) ion trap mass spectrometer. The samples are dissolved in water-acetonitrile (50:50) with 0.1% formic acid.

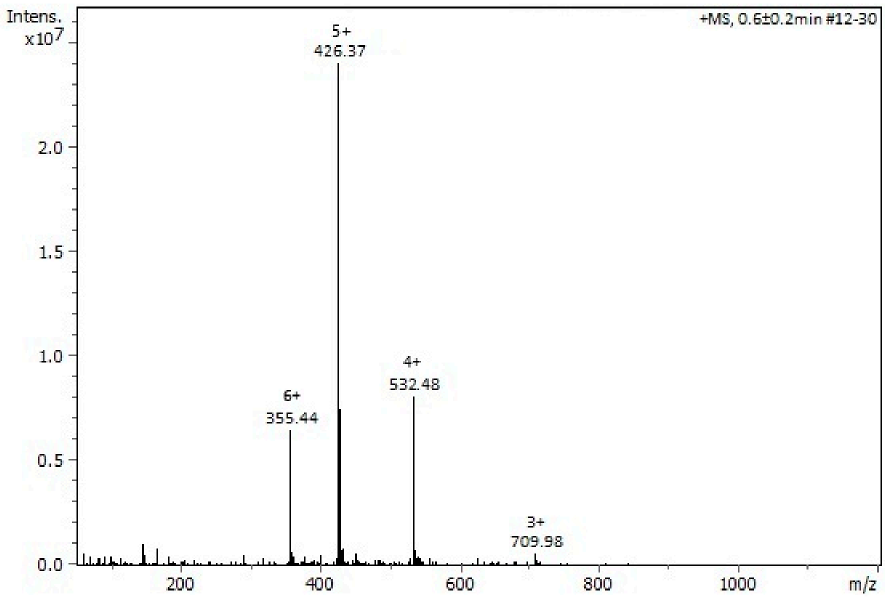

Figure S37. MS Spectrum of Ac-Pen12(1Nal)(Cf) 1a. The identity of the peptide conjugate was determined using Bruker Amazon SL (Germany) ion trap mass spectrometer. The samples are dissolved in water-acetonitrile (50:50) with 0.1% formic acid.

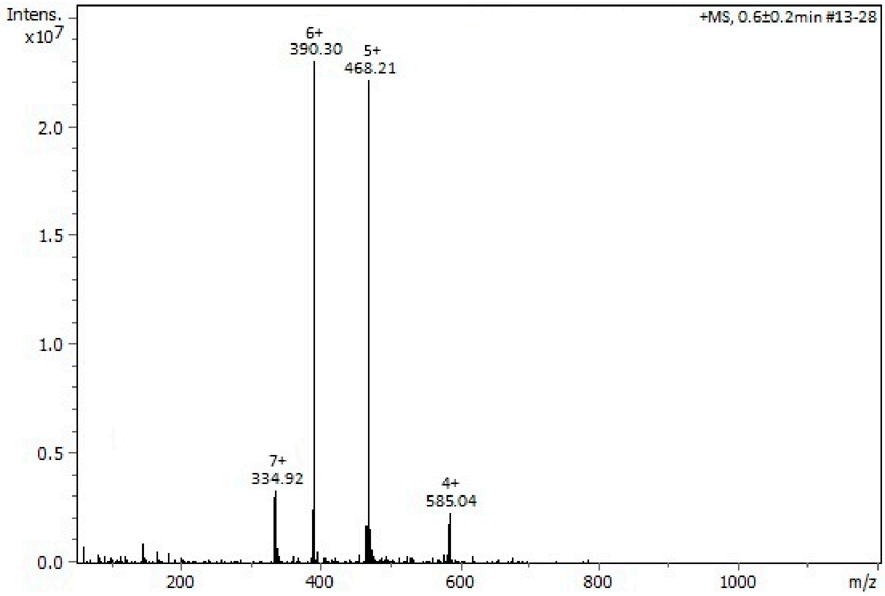

Figure S38. MS Spectrum of Dabcyl-Pen12(1Nal)(Cf) 1b. The identity of the peptide conjugate was determined using Bruker Amazon SL (Germany) ion trap mass spectrometer. The samples are dissolved in water-acetonitrile (50:50) with 0.1% formic acid.

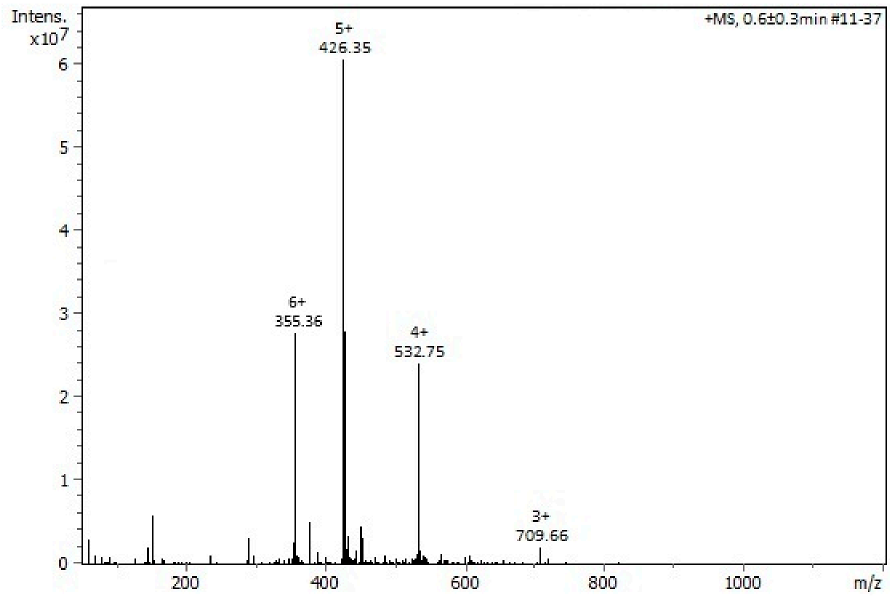

Figure S39. MS Spectrum of Ac-Pen12(1Nal)(Cf) 2a. The identity of the peptide conjugate was determined using Bruker Amazon SL (Germany) ion trap mass spectrometer. The samples are dissolved in water-acetonitrile (50:50) with 0.1% formic acid.

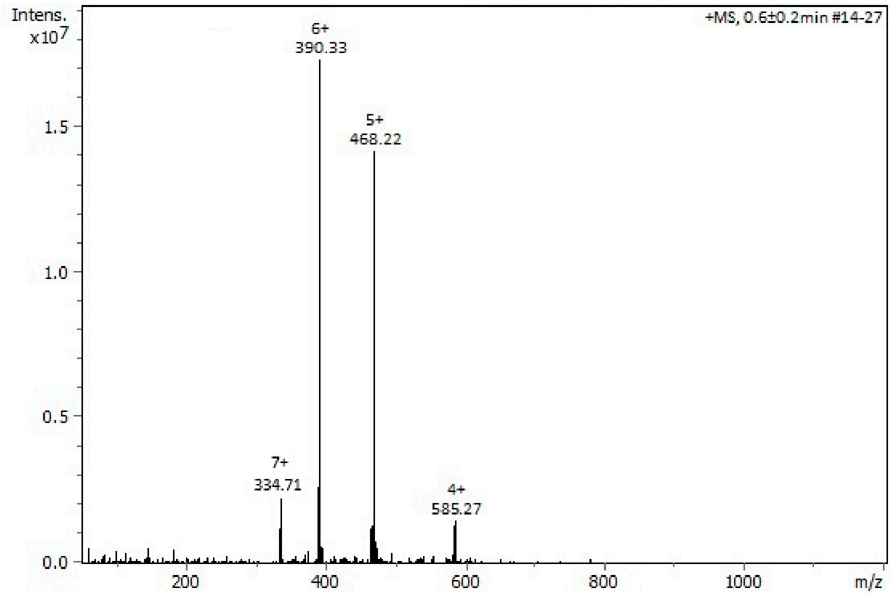

Figure S40. MS Spectrum of Dabcyl-Pen12(1Nal)(Cf) 2b. The identity of the peptide conjugate was determined using Bruker Amazon SL (Germany) ion trap mass spectrometer. The samples are dissolved in water-acetonitrile (50:50) with 0.1% formic acid.

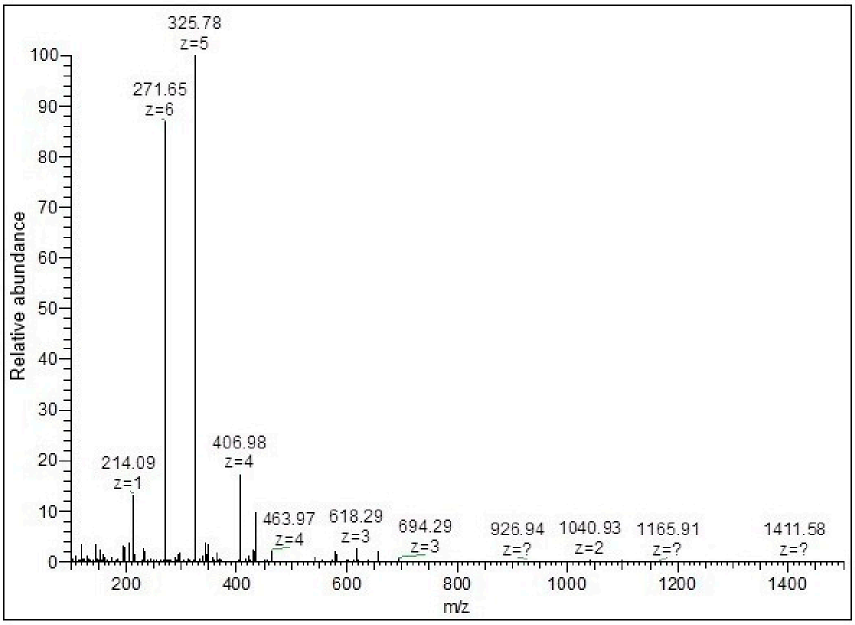

Figure S41. MS Spectrum of Cf-(Arg)$_8$ The identity of the peptide conjugate was determined using Bruker Daltonics Esquire 3000 plus (Germany) ion trap mass spectrometer. The samples are dissolved in water-acetonitrile (50:50) with 0.1% formic acid.

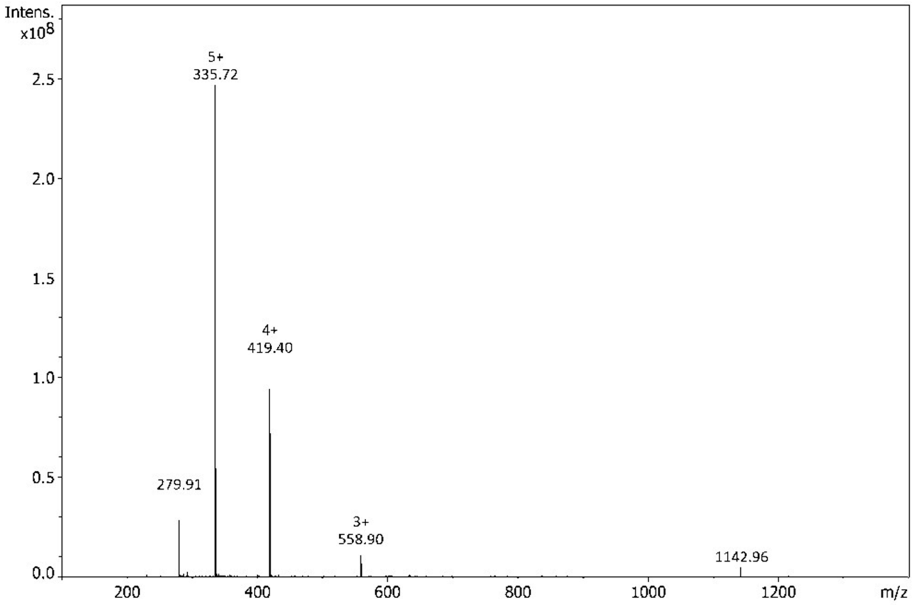

Figure S42. MS Spectrum of NH_2-Pen12. The identity of the peptide conjugate was determined using Bruker Amazon SL (Germany) ion trap mass spectrometer. The samples are dissolved in water-acetonitrile (50:50) with 0.1% formic acid.

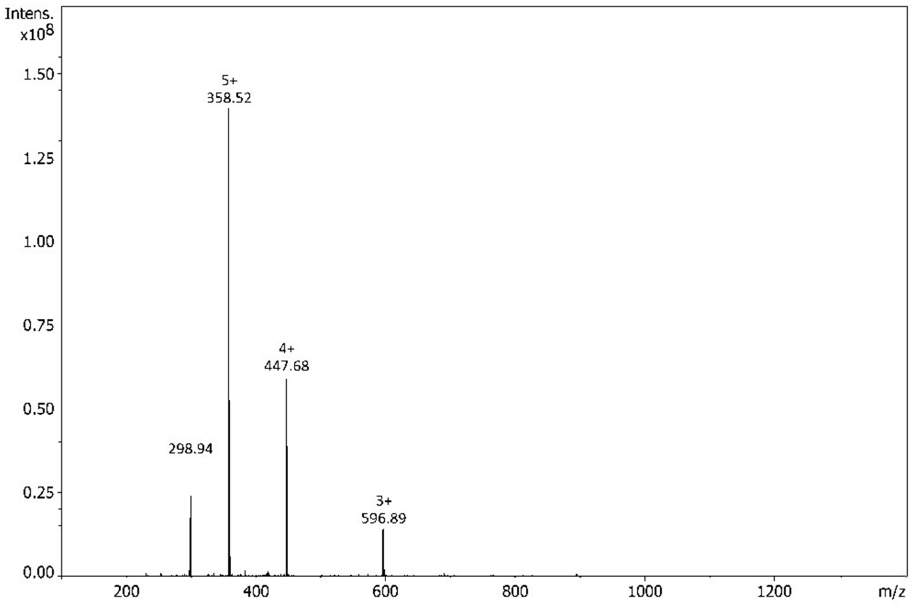

Figure S43. MS Spectrum of NH_2-Pen12(3Nal). The identity of the peptide conjugate was determined using Bruker Amazon SL (Germany) ion trap mass spectrometer. The samples are dissolved in water-acetonitrile (50:50) with 0.1% formic acid.

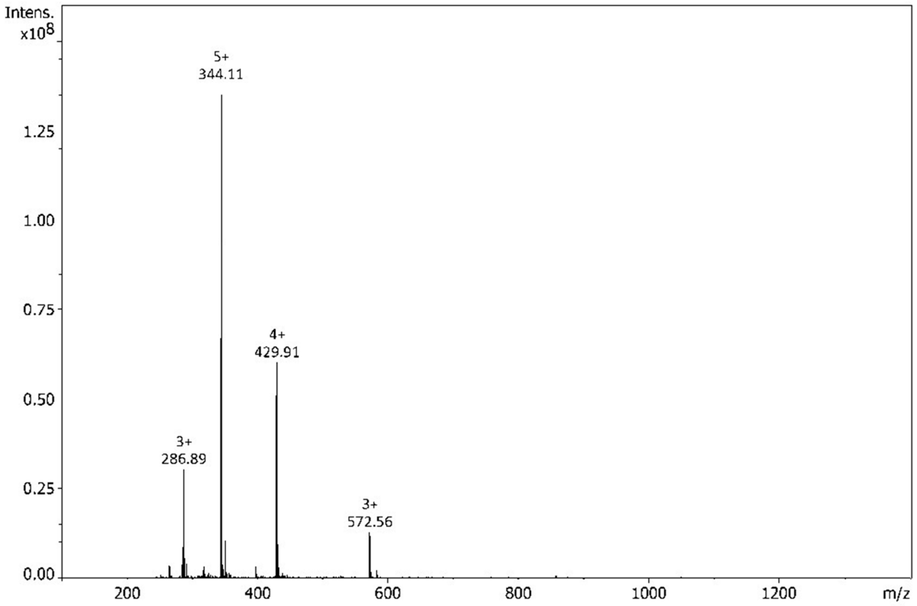

Figure S44. MS Spectrum of NH_2-Pen12(3TIC). The identity of the peptide conjugate was determined using Bruker Amazon SL (Germany) ion trap mass spectrometer. The samples are dissolved in water-acetonitrile (50:50) with 0.1% formic acid.

3. Confocal microscopy to assess intracellular distribution

We have visualized the intracellular localization of the labeled peptides using confocal laser scanning microscopy at 12.5 μM peptide concentration (based on the flow cytometry profile), and A-431 cells were incubated for 90 min. Representative images are presented (blue: Hoechst, nuclear dye; green: Cf-labelled peptide signal; red: Lysotracker Deep Red). Fixed A-431 cells were studied, and the treatment, staining, and fixation process were

carefully optimized to assess comparable greyscale values corresponding to green (intensity of Cf) and red (intensity of LysoTracker Deep Red dye) signals.

In accordance with the fluorescent signals, the peptides accumulated in the cytosol mainly in a punctuate manner, and partially co-localized with the lysosomes. The co-localization analysis was made based on our previous paper [51].

4. Internalization and intracellular localization analysis based on confocal microscopic images

The Cf-labelled peptides' cellular internalization was captured by a confocal microscope. The intracellular localization was assessed using the following approaches:

Analysis of the unprocessed images with LasX software of Leica SP8. (Figure S45 – Table S1, Figure S47 - Table S3, Figure S49 - Table S5, Figure S51 - Table S7, Figure S53 - Table S9)

"In-house Python-based" image analysis. (Figure S46 – Table S2, Figure S48 - Table S4, Figure S50 - Table S6, Figure S52 - Table S8, Figure S54 - Table S10)

The unprocessed images (blue, green, red) will allow us to track the peptide uptake separately according to cellular niches (nucleus, lysosome) by color. First, we normalize each pixel intensity value [44] and then flatten the images [44] (reduce the two-dimensional array – the pixel coordinates – into a one-dimensional list of normalized intensity values) for each channel. These lists of values were used to calculate the Pearson correlations [45] (red-green lysosomal, blue-green nuclear).

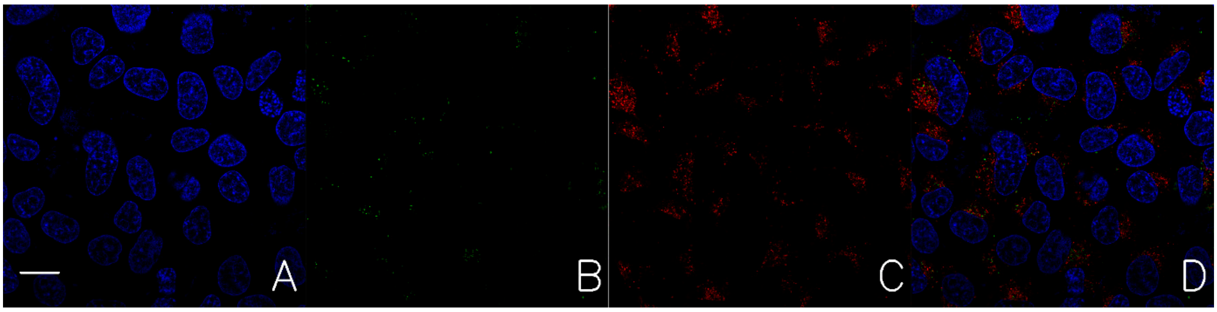

Figure S45. Internalization of peptide Cf-Pen captured by confocal laser scanning microscopy using unprocessed images. Cells were incubated for 90 minutes with peptide (c=12.5 μM). Nuclei were stained with Hoechst 33342, blue (A); Cf labelled peptide, green (B); lysosomes were labelled with LysoTracker Deep Red, red (C). Cells were examined using a Leica SP8 microscope with an HC PL APO CS2 63x/1.40 OIL objective and the hybrid detector. The scale bar represents 20 μm.

Table S1. Co-localization analysis using single-channel confocal "tif" images (unprocessed images from Leica LasX software).

Pearson Correlation (Red-Green)[a]	Pearson Correlation (Blue-Green)[b]	Lcoeff (Red)[c]	Lcoeff (Blue)[d]	Average Intensity (Green in Red Uptake)[e]	Average Intensity (Green in Blue Uptake)[f]	Average Intensity (Green)[g]
0,2008	-0,0345	0,0746	0,0325	17,7600	17,5600	10,4300

The „in-house Python-based" method calculates the "Pearson Correlations," the Lcoeff (Red, Blue), and the average intensity values of green channel intensities. We created categories by filtering the channels based on varying criteria. We also count the sum of the number of pixels of each category. We developed metrics based on channel overlap and used these metrics to calculate the Lcoeff (Red, Blue).

[a]Value calculated by a Python module from the flattened red and green channels. Relative value (-1-1) that reflects the linear correlation of two sets of the intensity values of the red and green channel pixels

[b]Value calculated by a Python module [44] from the flattened red and green channels. Relative value (-1-1) that reflects the linear correlation of two sets of the intensity values of the blue and green channel pixels

[c]Co-localization value calculated from red and green channel pixel counts employing the developed equation

[d]Co-localization value calculated from blue and green channel pixel counts employing the developed equation

[e]Calculated average intensity of peptides co-localized with lysosomes

[f]Calculated average intensity of peptides co-localized with nuclei

[g]Calculated average intensity of peptides in cell cytosol/cytoplasm

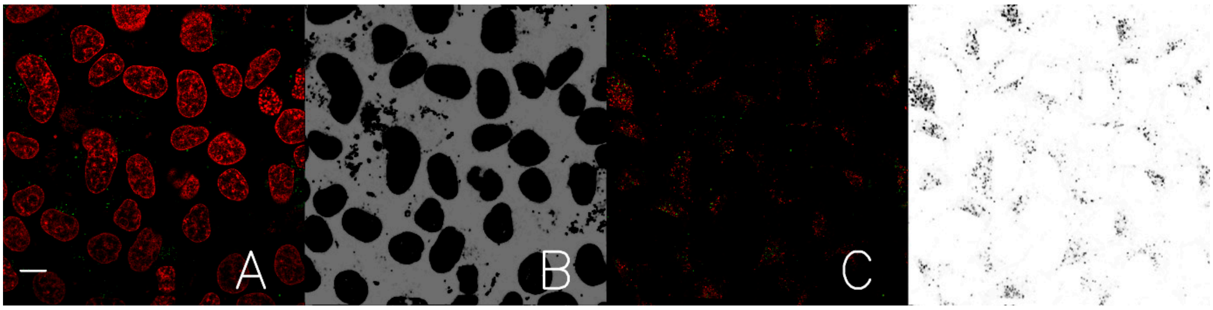

Figure S46. Costes' mask and spatial Pearson images were retrieved from Figure S45. with ImageJ using JACoP. [48,49] Costes' Mask: ImageJ JACoP performs Costes' automatic threshold analysis. The created mask highlights the co-localized regions of the compared channels. [48,49] Costes's Mask, blue and green channels compared from Figure S45. partD (A); Spatial Pearson map, blue and green channels compared from Figure S45. partD (B); Costes' Mask, red and green channels compared from Figure S45. partD (C); Spatial Pearson map, red and green channels compared from Figure S45. partD (D).

Table S2. Based on our previous studies [46,47], we have also evaluated the co-localization using ImageJ JACoP. [48,49]

		Red-Green	Blue-Green
**Pearson Coefficient**[a]		0.2	-0.034
**Manders Coefficients**[b]	M1	0.219	0.009
(original)	M2	0.947	0.627

[a]This is a linear equation that uses linear regression to calculate the relationship between the intensities of two images. 1 value stands for complete correlation, and -1 stands for complete negative correlation, while zero stands for no correlation at all

[b]Manders' overlap coefficient is based on Pearson but takes out the average intensities. The values range from 0 to 1. M1 and M2 are relative values between the sum intensity of the channel, whereas the other channel has a value above zero compared to the total sum intensity of the channel. [48,49]

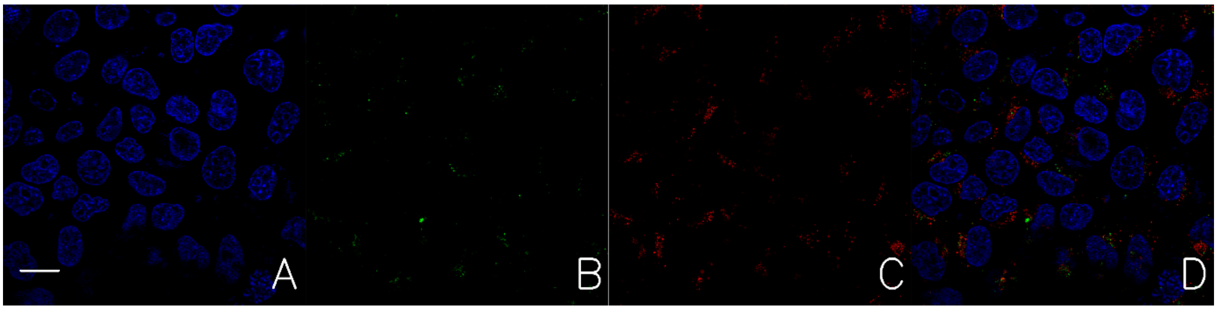

Figure S47. Internalization of peptide Cf-Pen12 captured by confocal laser scanning microscopy using unprocessed images. Cells were incubated for 90 minutes with peptide (c=12.5 μM). Nuclei were stained with Hoechst 33342, blue (A); Cf labelled peptide, green (B); lysosomes were labelled with LysoTracker Deep Red, red (C). Cells were examined using a Leica SP8 microscope with an HC PL APO CS2 63x/1.40 OIL objective and the hybrid detector. The scale bar represents 20 μm.

Table S3. Co-localization analysis using single-channel confocal "tif" images (unprocessed images from Leica LasX software).

**Pearson Correlation** (Red-Green)[a]	**Pearson Correlation** (Blue-Green)[b]	**L_{coeff}** (Red)[c]	**L_{coeff}** (Blue)[d]	**Average Intensity** (Green in Red Uptake)[e]	**Average Intensity** (Green in Blue Uptake)[f]	**Average Intensity** (Green)[g]
0,3211	-0,0503	0,1723	0,0842	11,8500	10,9100	3,8800

The „in-house Python-based" method calculates the "Pearson Correlations," the Lcoeff (Red, Blue), and the average intensity values of green channel intensities. We created categories by filtering the channels based on varying criteria. We also count the sum of the number of pixels of each category. We developed metrics based on channel overlap and used these metrics to calculate the Lcoeff (Red, Blue).

[a]Value calculated by a Python module from the flattened red and green channels. Relative value (-1-1) that reflects the linear correlation of two sets of the intensity values of the red and green channel pixels

[b]Value calculated by a Python module from the flattened red and green channels. Relative value (-1-1) that reflects the linear correlation of two sets of the intensity values of the blue and green channel pixels

[c]Co-localization value calculated from red and green channel pixel counts employing the developed equation

[d]Co-localization value calculated from blue and green channel pixel counts employing the developed equation

[e]Calculated average intensity of peptides co-localized with lysosomes

[f]Calculated average intensity of peptides co-localized with nuclei

[g]Calculated average intensity of peptides in cell cytosol/cytoplasm

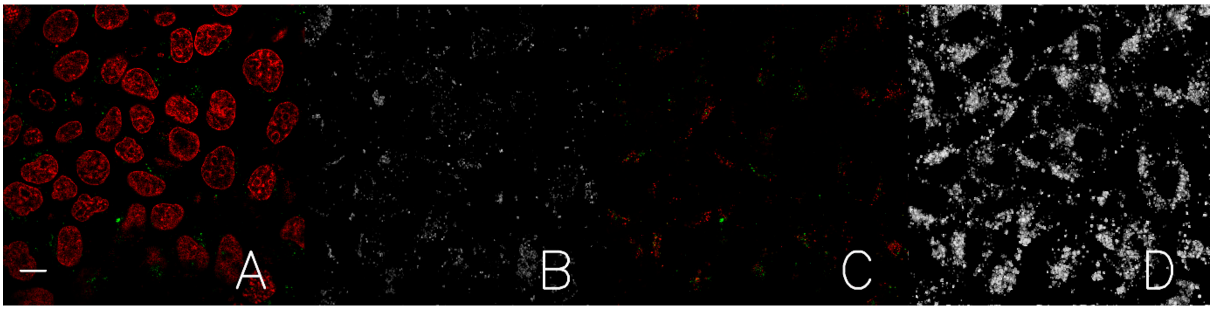

Figure S48. Costes' mask and spatial Pearson images were retrieved from Figure S47. with ImageJ using JACoP. [48,49] Costes' Mask: ImageJ JACoP performs Costes' automatic threshold analysis. The created mask highlights the co-localized regions of the compared channels. [48,49] Costes's Mask, blue and green channels compared from Figure S47. partD (A); Spatial Pearson map, blue and green channels compared from Figure S48. partD (B); Costes' Mask, red and green channels compared from Figure S47. partD (C); Spatial Pearson map, red and green channels compared from Figure S47. partD (D).

Table S4. Based on our previous studies [46,47], we have also evaluated the co-localization using ImageJ JACoP. [48,49]

		Red-Green	Blue-Green
**Pearson Coefficient**[a]		0.321	-0.05
**Manders Coefficients**[b]	M1	0.457	0.037
(original)	M2	0.96	0.526

[a]This is a linear equation that uses linear regression to calculate the relationship between the intensities of two images. 1 value stands for complete correlation, and -1 stands for complete negative correlation, while zero stands for no correlation at all

[b]Manders' overlap coefficient is based on Pearson but takes out the average intensities. The values range from 0 to 1. M1 and M2 are relative values between the sum intensity of the channel, whereas the other channel has a value above zero compared to the total sum intensity of the channel. [48,49]

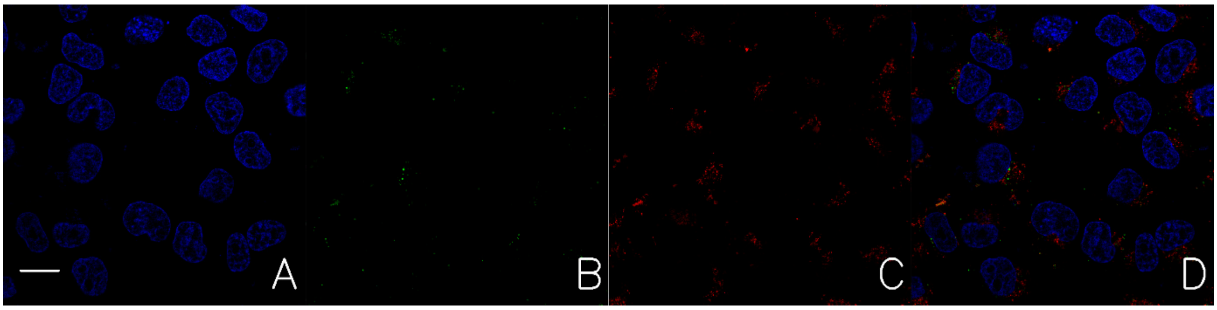

Figure S49. Internalization of peptide Ac-Pen-K(Cf) captured by confocal laser scanning microscopy using unprocessed images. Cells were incubated for 90 minutes with peptide (c=12.5 µM). Nuclei were stained with Hoechst 33342, blue (A); Cf labelled peptide, green (B); lysosomes were labelled with LysoTracker Deep Red, red (C). Cells were examined using a Leica SP8 microscope with an HC PL APO CS2 63x/1.40 OIL objective and the hybrid detector. The scale bar represents 20 µm.

Table S5. Co-localization analysis using single-channel confocal "tif" images (unprocessed images from Leica LasX software).

**Pearson Correlation** (Red-Green)[a]	**Pearson Correlation** (Blue-Green)[b]	**Lcoeff** (Red)[c]	**Lcoeff** (Blue)[d]	**Average Intensity** (Green in Red Uptake)[e]	**Average Intensity** (Green in Blue Uptake)[f]	**Average Intensity** (Green)[g]
0,2283	-0,0245	0,0962	0,0262	18,0400	17,3800	11,5100

The „in-house Python-based" method calculates the "Pearson Correlations," the Lcoeff (Red, Blue), and the average intensity values of green channel intensities. We created categories by filtering the channels based on varying criteria. We also count the sum of the number of pixels of each category. We developed metrics based on channel overlap and used these metrics to calculate the Lcoeff (Red, Blue).

[a]Value calculated by a Python module from the flattened red and green channels. Relative value (-1-1) that reflects the linear correlation of two sets of the intensity values of the red and green channel pixels

[b]Value calculated by a Python module from the flattened red and green channels. Relative value (-1-1) that reflects the linear correlation of two sets of the intensity values of the blue and green channel pixels

[c]Co-localization value calculated from red and green channel pixel counts employing the developed equation

[d]Co-localization value calculated from blue and green channel pixel counts employing the developed equation

[e]Calculated average intensity of peptides co-localized with lysosomes

[f]Calculated average intensity of peptides co-localized with nuclei

[g]Calculated average intensity of peptides in cell cytosol/cytoplasm

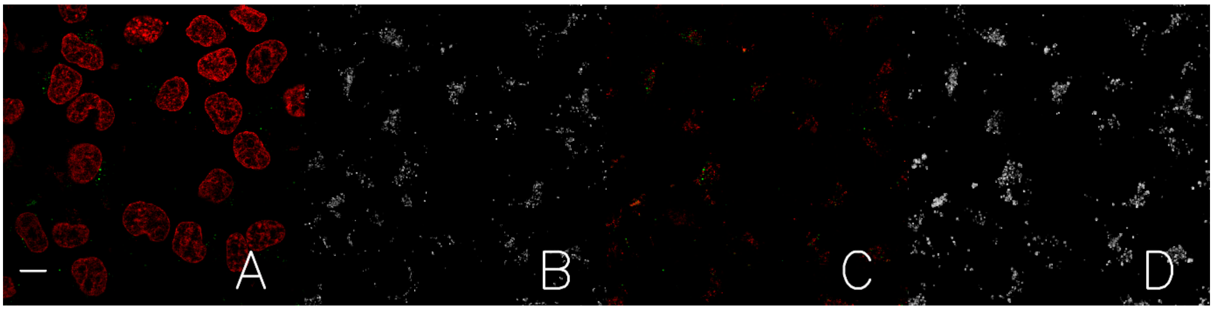

Figure S50. Costes' mask and spatial Pearson images were retrieved from Figure S49. with ImageJ using JACoP. [48,49] Costes' Mask: ImageJ JACoP performs Costes' automatic threshold analysis. The created mask highlights the co-localized regions of the compared channels. [48,49] Costes's Mask, blue and green channels compared from Figure S49. partD (A); Spatial Pearson map, blue and green channels compared from Figure S49. partD (B); Costes' Mask, red and green channels compared from Figure S49. partD (C); Spatial Pearson map, red and green channels compared from Figure S49 partD (D).

Table S6. Based on our previous studies [46,47], we have also evaluated the co-localization using ImageJ JACoP. [48,49]

		Red-Green	Blue-Green
**Pearson Coefficient**[a]		0.228	-0.024
**Manders Coefficients**[b]	M1	0.242	0.007
(original)	M2	0.886	0.414

[a]This is a linear equation that uses linear regression to calculate the relationship between the intensities of two images. 1 value stands for complete correlation, and -1 stands for complete negative correlation, while zero stands for no correlation at all

[b]Manders' overlap coefficient is based on Pearson but takes out the average intensities. The values range from 0 to 1. M1 and M2 are relative values between the sum intensity of the channel, whereas the other channel has a value above zero compared to the total sum intensity of the channel. [48,49]

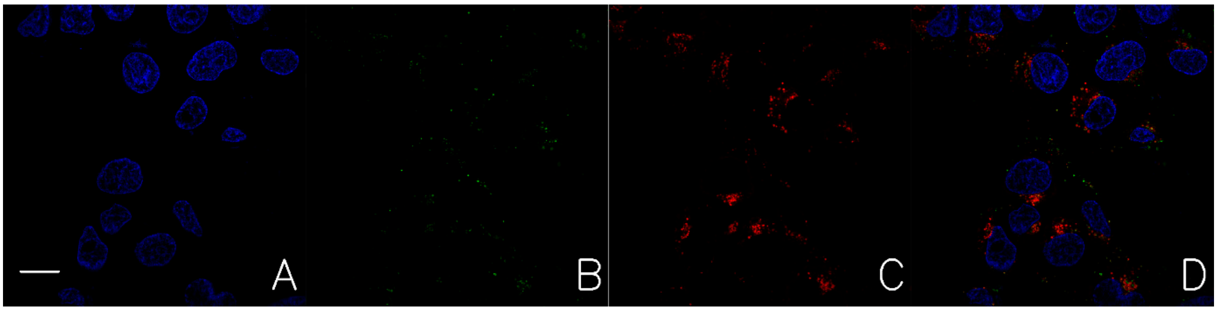

Figure S51. Internalization of peptide Dabcyl-Pen-K(Cf) captured by confocal laser scanning microscopy using unprocessed images. Cells were incubated for 90 minutes with peptide (c=12.5 μM). Nuclei were stained with Hoechst 33342, blue (A); Cf labelled peptide, green (B); lysosomes were labelled with LysoTracker Deep Red, red (C). Cells were examined using a Leica SP8 microscope with an HC PL APO CS2 63x/1.40 OIL objective and the hybrid detector. The scale bar represents 20 μm.

Table S7. Co-localization analysis using single-channel confocal "tif" images (unprocessed images from Leica LasX software).

**Pearson Correlation** (Red-Green)[a]	**Pearson Correlation** (Blue-Green)[b]	**L_{coeff}** (Red)[c]	**L_{coeff}** (Blue)[d]	**Average Intensity** (Green in Red Uptake)[e]	**Average Intensity** (Green in Blue Uptake)[f]	**Average Intensity** (Green)[g]
0,3881	-0,0266	0,2550	0,1215	11,0900	10,1100	3,9800

The „in-house Python-based" method calculates the "Pearson Correlations," the Lcoeff (Red, Blue), and the average intensity values of green channel intensities. We created categories by filtering the channels based on varying criteria. We also count the sum of the number of pixels of each category. We developed metrics based on channel overlap and used these metrics to calculate the Lcoeff (Red, Blue).

[a]Value calculated by a Python module from the flattened red and green channels. Relative value (-1-1) that reflects the linear correlation of two sets of the intensity values of the red and green channel pixels

[b]Value calculated by a Python module from the flattened red and green channels. Relative value (-1-1) that reflects the linear correlation of two sets of the intensity values of the blue and green channel pixels

[c]Co-localization value calculated from red and green channel pixel counts employing the developed equation

[d]Co-localization value calculated from blue and green channel pixel counts employing the developed equation

[e]Calculated average intensity of peptides co-localized with lysosomes

[f]Calculated average intensity of peptides co-localized with nuclei

[g]Calculated average intensity of peptides in cell cytosol/cytoplasm

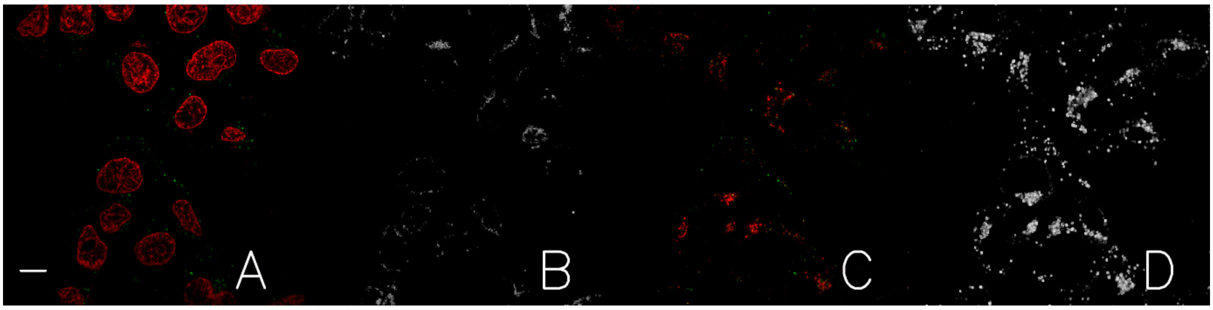

Figure S52. Costes' mask and spatial Pearson images were retrieved from Figure S51. with ImageJ using JACoP. [48,49] Costes' Mask: ImageJ JACoP performs Costes' automatic threshold analysis. The created mask highlights the co-localized regions of the compared channels. [48,49] Costes's Mask, blue and green channels compared from Figure S51. partD (A); Spatial Pearson map, blue and green channels compared from Figure S51. partD (B); Costes' Mask, red and green channels compared from Figure S51. partD (C); Spatial Pearson map, red and green channels compared from Figure S51. partD (D).

Table S8. Based on our previous studies [46,47], we have also evaluated the co-localization using ImageJ JACoP. [48,49]

		Red-Green	Blue-Green
**Pearson Coefficient**[a]		0.388	-0.026
**Manders Coefficients**[b]	M1	0.57	0.046
(original)	M2	0.884	0.362

[a]This is a linear equation that uses linear regression to calculate the relationship between the intensities of two images. 1 value stands for complete correlation, and -1 stands for complete negative correlation, while zero stands for no correlation at all

[b]Manders' overlap coefficient is based on Pearson but takes out the average intensities. The values range from 0 to 1. M1 and M2 are relative values between the sum intensity of the channel, whereas the other channel has a value above zero compared to the total sum intensity of the channel. [48,49]

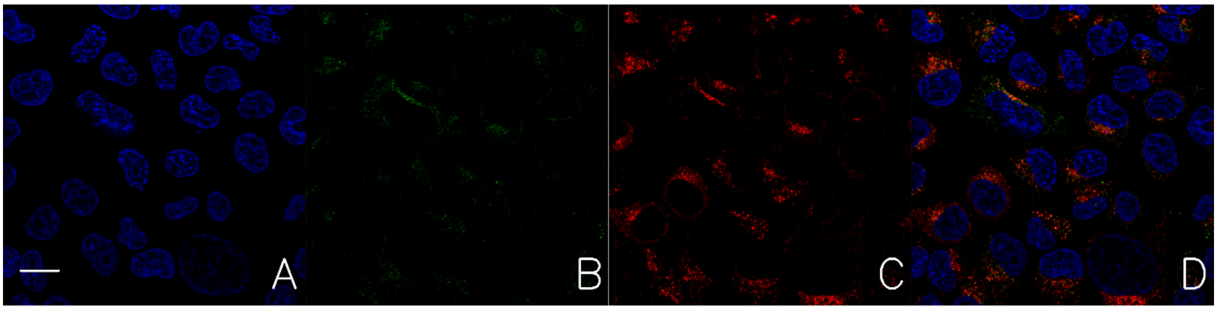

Figure S53. Internalization of peptide Dabcyl-Pen12-K(Cf) captured by confocal laser scanning microscopy using unprocessed images. Cells were incubated for 90 minutes with peptide (c=12.5 μM). Nuclei were stained with Hoechst 33342, blue (A); Cf labelled peptide, green (B); lysosomes were labelled with LysoTracker Deep Red, red (C). Cells were examined using a Leica SP8 microscope with an HC PL APO CS2 63x/1.40 OIL objective and the hybrid detector. The scale bar represents 20 μm.

Table S9. Co-localization analysis using single-channel confocal "tif" images (unprocessed images from Leica LasX software).

Pearson Correlation (Red-Green)[a]	Pearson Correlation (Blue-Green)[b]	L_{coeff} (Red)[c]	L_{coeff} (Blue)[d]	Average Intensity (Green in Red Uptake)[e]	Average Intensity (Green in Blue Uptake)[f]	Average Intensity (Green)[g]
0,5289	-0,1338	0,6641	0,3320	11,5600	10,5800	4,3000

The „in-house Python-based" method calculates the "Pearson Correlations," the Lcoeff (Red, Blue), and the average intensity values of green channel intensities. We created categories by filtering the channels based on varying criteria. We also count the sum of the number of pixels of each category. We developed metrics based on channel overlap and used these metrics to calculate the Lcoeff (Red, Blue).

[a]Value calculated by a Python module from the flattened red and green channels. Relative value (-1-1) that reflects the linear correlation of two sets of the intensity values of the red and green channel pixels

[b]Value calculated by a Python module from the flattened red and green channels. Relative value (-1-1) that reflects the linear correlation of two sets of the intensity values of the blue and green channel pixels

[c]Co-localization value calculated from red and green channel pixel counts employing the developed equation

[d]Co-localization value calculated from blue and green channel pixel counts employing the developed equation

[e]Calculated average intensity of peptides co-localized with lysosomes

[f]Calculated average intensity of peptides co-localized with nuclei

[g]Calculated average intensity of peptides in cell cytosol/cytoplasm

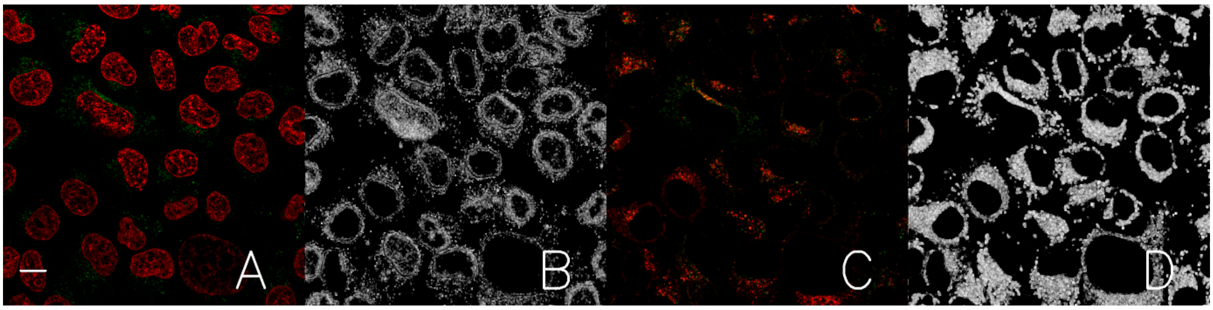

Figure S54. Costes' mask and spatial Pearson images were retrieved from Figure S53. with ImageJ using JACoP. [48,49] Costes' Mask: ImageJ JACoP performs Costes' automatic threshold analysis. The created mask highlights the co-localized regions of the compared channels. [48,49] Costes's Mask, blue and green channels compared from Figure S53. partD (A); Spatial Pearson map, blue and green channels compared from Figure S53. partD (B); Costes' Mask, red and green channels compared from Figure S53. partD (C); Spatial Pearson map, red and green channels compared from Figure S53. partD (D).

Table S10. Based on our previous studies [46,47], we have also evaluated the co-localization using ImageJ JACoP. [48,49]

		Red-Green	Blue-Green
**Pearson Coefficient**[a]		0.528	-0.133
**Manders Coefficients**[b]	M1	0.826	0.237
(original)	M2	0.885	0.37

[a]This is a linear equation that uses linear regression to calculate the relationship between the intensities of two images. 1 value stands for complete correlation, and -1 stands for complete negative correlation, while zero stands for no correlation at all

[b]Manders' overlap coefficient is based on Pearson but takes out the average intensities. The values range from 0 to 1. M1 and M2 are relative values between the sum intensity of the channel, whereas the other channel has a value above zero compared to the total sum intensity of the channel. [48,49]

5. Electronic circular dichroism spectroscopy

The secondary structures of three selected peptides Pen12, Pen12(3Nal) and Pen12(3TIC) (Table S11.) were examined by electronic circular dichroism (ECD) spectroscopy. Stock solutions with a concentration of 0.1 mg/ml and pH= 7.4 were prepared, then these were diluted fourfold before the measurements. CD spectra of the peptides were recorded at 5°C, 25°C and 85°C between 190-250 nm with a Jasco J-1500 CD Spectrometer (1 mm cuvette, 50 nm/scan).

Table S11. Characterization of peptides studied by ECD

Abbreviation	Sequence	M_w (g/mol)
Pen12	*NH₂*-RQIKIWFRKWKK-*NH₂*	1715.06
Pen12(3Nal)	*NH₂*-RQIKI-*Nal*-*Nal*-RK-*Nal*-KK-*NH₂*	1787.09
Pen12(3TIC)	*NH₂*-RQIKI-*TIC*-*TIC*-RK-*TIC*-KK-*NH₂*	1673.04

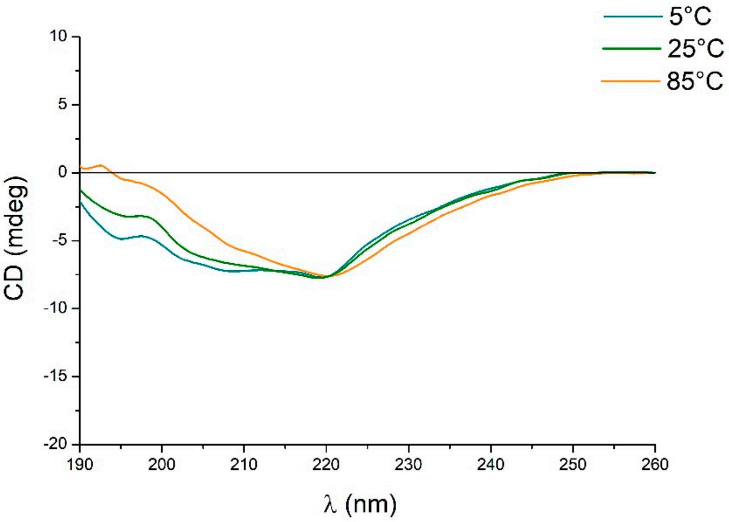

Figure S55. CD spectrum of Pen12 (*H*-RQIKIWFRKWKK-*NH₂*) at different temperatures

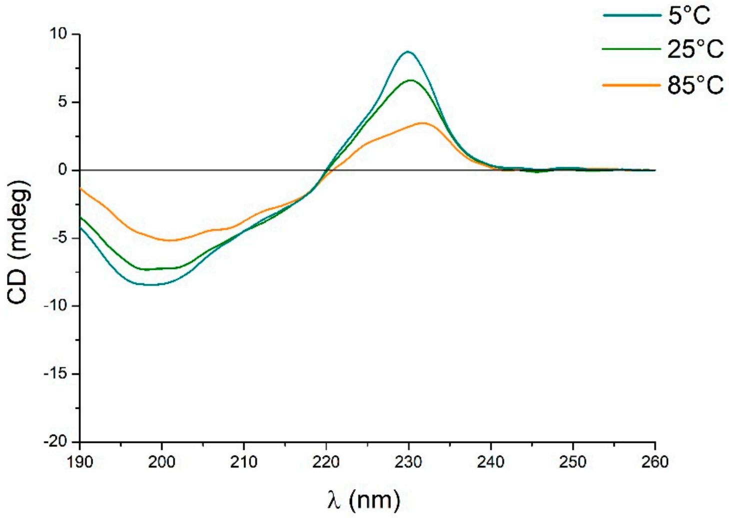

Figure S56. CD spectrum of Pen12(3Nal) (*H*-RQIKI-*Nal*-*Nal*-RK-*Nal*-KK-*NH*2) at different temperatures

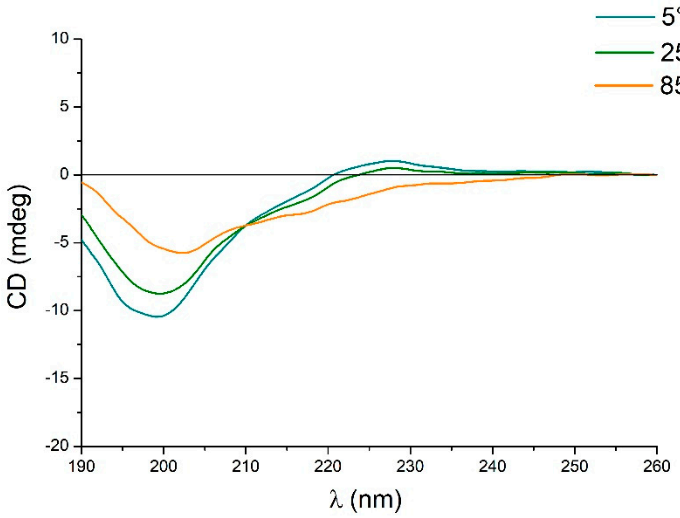

Figure S57. CD spectrum of Pen12(3TIC) (*H*-RQIKI-*TIC*-*TIC*-RK-*TIC*-KK-*NH*2) at different temperatures

6. Cytotoxicity of peptides

The cytotoxicity of prepared penetratin derivative was assessed by measuring the ratio of live cells during flow cytometry.

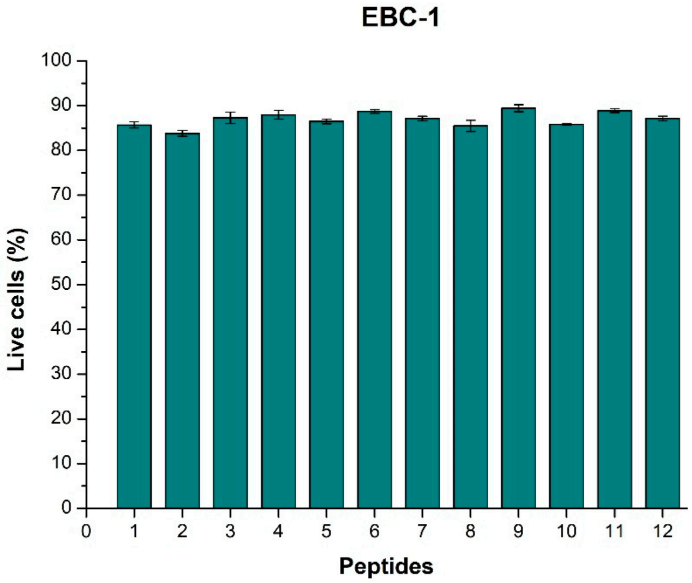

Figure S58. Determination of the cytotoxicity of peptide conjugates by assessing the percentage of live cells by flow cytometry. EBC-1 cells were treated with peptides at 5 μM for 90 min. The numbers

indicate the following: 1- water control, 2- Cf-Pen, 3- Cf-Pen12, 4- Cf-Pen(3Nal), 5- Cf-Pen12(3Nal), 6- Cf-Pen(3TIC), 7- Cf-Pen12(3TIC), 8- Ac-Pen12(Cf), 9- Dabcyl-Pen12(Cf), 10- Ac-Pen12(3Nal)(Cf), 11- Dabcyl-Pen12(3Nal)(Cf), 12- Cf-(Arg)$_8$.

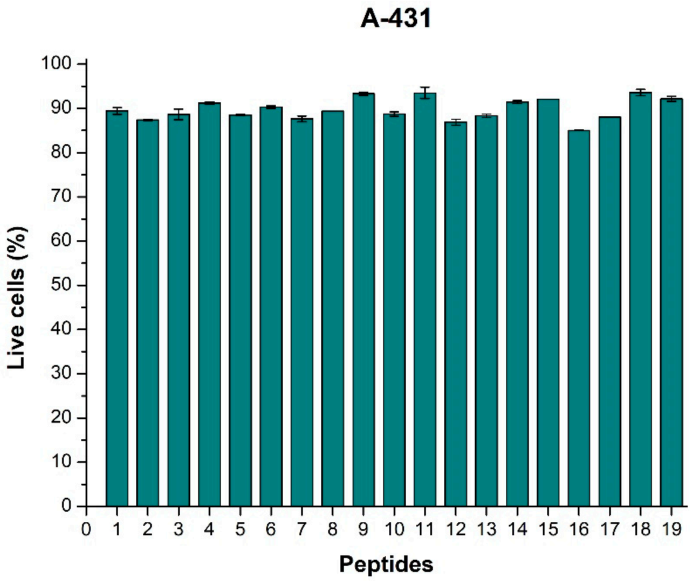

Figure S59. Determination of the cytotoxicity of peptide conjugates by assessing the percentage of live cells by flow cytometry. A-431 cells were treated with peptides at 10 μM for 90 min. The numbers indicate the following: 1- water control, 2- Cf-Pen, 3- Cf-Pen12, 4- Cf-Pen(3Nal), 5- Cf-Pen12(3Nal), 6- Cf-Pen(3TIC), 7- Cf-Pen12(3TIC), 8- Ac-Pen-K(Cf), 9- Dabcyl-Pen-K(Cf), 10- Ac-Pen12-K(Cf), 11- Dabcyl-Pen12-K(Cf), 12- Ac-Pen12(Cf), 13- Dabcyl-Pen12(Cf), 14- Ac-Pen12(3Nal)(Cf), 15- Dabcyl-Pen12(3Nal)(Cf), 16- Ac-Pen12(1Nal)(Cf) 1a, 17- Ac-Pen12(1Nal)(Cf) 2a, 18- Dabcyl-Pen12(1Nal)(Cf) 1b, 19- Dabcyl-Pen12(1Nal)(Cf) 2b.
